# Recreational Drug use at Sports Events in the US and UK

**DOI:** 10.1177/01937235251387699

**Published:** 2025-10-21

**Authors:** Martha Newson, Linus Peitz, Mollie Ruler

**Affiliations:** 1 4918 University of Greenwich, London, UK; 2 6396 University of Oxford, Oxford, UK; 3 2240 University of Kent, Canterbury, UK; 4 7423 University of Southampton, Southampton, UK

**Keywords:** recreational drugs, sports fans, cocaine, cannabis, fan behavior

## Abstract

Recreational drug use among sports fans has received relatively little scholarly attention. Nonetheless, understanding this landscape is crucial to better understand fan behaviors and attitudes, as well as to support fan communities through effective harm reduction and educational initiatives. Addressing this gap, we surveyed fans in the US (football, baseball, basketball, ice hockey) and the UK (soccer, rugby, cricket) to assess the prevalence, correlates, contexts, and motivations behind drug use at major sporting events (*N* = 2,556). Fans reported more drug use than the general population, with significantly more use in the US (22.9%) than in the UK (6.5%), where there was more alcohol consumption. There were no significant differences for drug use at games between sports in the US, yet in the UK, soccer (8.9%) and rugby (8.3%) fans reported more use than cricket fans (2.2%). Drug types, motivations for use, and demographic correlates of use were broadly consistent across sports, whereas the role of collective identities was distinct according to national context. In the UK, team bonding was associated with both drug use and support of sanctions for drugs at games, aligning with a carnivalesque interpretation of fan behavior, where temporary suspension of broader social norms may coexist with internal group regulation. In the US, by contrast, team bonding was unrelated to drug-taking, with divergent effects on support for sanctions suggesting tensions between inclusive group norms and efforts to police in-group transgressions. Findings point to a need for tailored harm reduction and educational initiatives: we suggest that acknowledging drug use as part of fan culture could inform targeted interventions to reduce shame and better prioritize education, safety, and well-being within sports communities.

## Introduction

Recreational drug use has been increasing globally, with the United Nations suggesting a 23% increase over the past decade (UNODC, 2023). To effectively support educational initiatives that promote healthy attitudes toward drug use and ensure safe consumption practices, a crucial first step is to understand the recreational drug use landscape, including where they are used, the motivations behind their use, and the behaviors associated with them. In certain cultural contexts, recreational drug use has become more visible and, in some cases, more accepted. For instance, music festivals and the nighttime economy have shifted toward harm reduction strategies in the last two decades, including drug-testing services, even where substances remain illegal (Winstock et al., 2022). However, sports events have been slower to enter such public health conversations, despite their large crowds and intense emotional engagement. This is perhaps surprising since fan safety has been prioritized by stakeholders for decades (e.g., see Chalmers & Frosdick, 2011; Taylor & Toohey, 2011). While alcohol consumption at sporting events is widely recognized and studied (e.g., Pradhan et al., 2021), we sought to build on evidence that indicates higher-than-typical population use of other drugs among sports fans in the UK (Newson, 2021). In doing so, we argue that recreational drug use in sports settings deserves attention not only as a public health issue, but as a social phenomenon that reflects the cultural dynamics of fandom and group identity.

We propose the carnivalesque as a key theoretical lens for understanding recreational drug use at sports events (Bakhtin, [1968] 2020; Giulianotti, 1995; Pearson, 2012; Turner, 2013). Traditionally associated with spaces of social inversion, collective pleasure, and temporary norm-breaking, the carnivalesque helps explain how certain behaviors, such as drug consumption, may become normalized or even ritualized in these emotionally heightened contexts (Pearson, 2012). This perspective allows us to view drug use not merely as individual hedonism but as an act embedded within fan culture, community, and collective transgression. Supporting this carnivalesque framing, we also draw on identity fusion theory (Swann et al., 2009), which might help explain individual differences in fan attitudes and behaviors, related to the deep psychological ties they may have to their teams. Sports team identities have previously been shown to be a powerful factor for fan engagement (Melnick & Wann, 2011), wellbeing (Wann, 2006), aggression (Wann et al., 1999), or willingness to engage in illegal behaviors (Wann et al., 2001). Together, these frameworks offer a sociocultural interpretation of drug use that moves beyond deviance or risk models, enabling a richer understanding of drug use at sports events.

## Literature Review

### Sports, Drugs, and the Carnivalesque

The literature on recreational substance use at sports events has predominantly focused on alcohol consumption due to its association with aggressive fan behaviors (Ostrowsky, 2016; Wakefield & Wann, 2006). For instance, studies have found that major sporting events correlate with spikes in alcohol-related emergency room visits and assaults (Miller et al., 2013), with debates surrounding alcohol sales at international tournaments such as the FIFA World Cup spanning decades (Caetano et al., 2012; Pearson & Sale, 2011). Among soccer fans, alcohol consumption is far from all negative and can also facilitate a collective suspension of social norms, offering an escape from the rigid conventions of modern society (Pearson, 2012). This desire for the ‘carnivalesque’ is not unique to soccer but is perhaps particularly pronounced, giving rise to a moderate-sized but influential literature on the carnivalesque nature of soccer fandom (Giulianotti, 1995, 2011; Hoy, 1994; Pearson, 2012; Turner, 2013).

Originating from Bakhtin's original work on ritual and the inversion of norms ([1968] 2020), the carnivalesque can manifest in various ways, from uninhibited sociability to profane language and even a complete social breakdown with violent disorder, all of which are rooted in social connection and solidarity with one's fellow ritual attendees. Giulianotti's (1995) seminal ethnographic work conceptualized soccer crowds as liminal spaces where everyday hierarchies are suspended and new, temporary identities emerge; here, traditional structures of decorum, authority, and class can dissolve. While Giulianotti (1991, 1995) draws primarily on Bakhtin's carnivalesque, his ethnographic observations also resonate with Turner's (1975) notion of *communitas*, the spontaneous solidarity that emerges in ritual spaces of inversion and liminality. In such spaces, behaviors that would be deemed inappropriate or deviant in everyday life are temporarily legitimized through their embeddedness in fan rituals, e.g., public intoxication, chanting, cursing, or even fighting. These rituals are not necessarily anarchic; rather, they follow an alternative moral logic rooted in collective identity and the symbolic function of the matchday event. In this framing, the carnivalesque is not merely about disorder, but about the creative inversion of order, where fans momentarily reclaim agency and community in contrast to bureaucratized, commodified everyday life.

This carnivalesque framework also helps explain why drug and alcohol consumption at matches is not always viewed as problematic by participants. As Pearson (2012) and Bandura et al. (2024) argue, substances like alcohol and, increasingly, drugs play a functional role in enhancing the euphoric, immersive, and transgressive qualities of the sporting spectacle. Here, intoxication is not deviant *per se* but becomes part of the shared semiotics of fandom, a social cue signaling participation in the ritual. Giulianotti's work remains particularly relevant in this regard, as he emphasizes how soccer fandom incorporates not only rebellion against external norms but also the internal reproduction of fan-based traditions, including drinking cultures and performative behavior. This dual structure, both subversive and structured, resonates strongly with the way carnivalesque behaviors unfold at sports events. Such rituals are deeply meaningful for participants, providing an outlet for identity, emotional intensity, and resistance, all of which are often expressed through embodied, sometimes intoxicant-fueled, celebration or confrontation.

Importantly, there is mounting evidence that fans use not only alcohol but also illicit substances to reclaim this sense of autonomy, with the use of high-risk controlled drugs (e.g., cocaine) potentially emerging as expressions of the carnivalesque (Bandura et al., 2024). Epidemiological studies from the US that analyze traces of drugs in wastewater from public facilities during and after games suggest that a variety of drugs, particularly cocaine, are consumed at college sporting events, including basketball and football, pointing to substantial on-site consumption (Lemas et al., 2021; Montgomery et al., 2021). Similar investigations further highlighted drug use among UK rugby fans, adding to growing concerns about its role in fan behavior (Buckland, 2024).

Public awareness of drug use at sporting events has grown in the UK, in part driven by safety concerns and the perceived link between illicit drug use and disorder. Even sports that are typically associated with family-friendly stadium atmospheres, such as cricket, have started to connect rare instances of antisocial crowd behavior with recreational drug use (Ammon, 2022), but large-scale data is yet to be gathered, let alone causal pathways established. Cocaine use among British soccer fans has come under particular scrutiny in the press and has also been associated with heightened aggression toward rival supporters in a correlational study, with 1% of fans reporting using cocaine in stadia and 1 in 3 witnessing others using it in an online study (Newson, 2021). Whether the combination of cocaine and alcohol exacerbates violent tendencies remains contested (Pennings et al., 2002; Redhead, 1993, 1997; van Amsterdam & van den Brink, 2023), and there are nations where little to no alcohol is consumed during sports games, but violence is still extreme, including deaths from fan violence – such as Indonesia, a Muslim majority nation where substance misuse and alcohol consumption is relatively low, but aggressive fan disorder is relatively high (Newson et al., 2024).

That said, the infiltration of the nighttime economy into sports is neither new nor particularly surprising given the population, opportunities for economic trade, and feelings of permissiveness (Ayres & Treadwell, 2012). However, the available evidence concerning drug use and the motivations for taking drugs in sports contexts is limited. Most studies focus on specific substances, sports, or events, and often rely on small sample sizes. We aim to address these issues by working with fans of the UK's and the US’ major sports using self-reported surveys targeting any recreational drug use in sports fandom.

We recognize that the carnivalesque atmosphere that many fans seek can be achieved in part by the consumption of drugs or the associated cultures drugs bring to a sport (Bandura et al., 2024; Pearson, 2012) but that other factors, including fan characteristics, social identities, and demographics, also play a role in how drugs may or may not prolificate. Such factors can be reflective of distinct fan cultures and populations, and possibly explain differences between sports contexts (e.g., in the UK, where sports are historically associated with socio-political backgrounds) or they could indicate correlates of substance use that are reflective of broader norms and behaviors in sports communities (e.g., prohibition of alcohol in the US and restricted use at sports events).

### Socio-Demographic Factors

Previous literature that captures drug use among sports fans quantitatively is limited, and therefore, relatively little is known about demographic patterns of drug use among fans, which was one of the motivations to pursue our work. There is some survey research with soccer fans (e.g., Newson, 2021), indicating that certain demographic drug use patterns from the general population are also reflected among sports fans: for instance, males consume more, and at higher risk levels (Cuttler et al., 2016; Wagner & Anthony, 2007). This aligns with related evidence that alcohol consumption is higher amongst male sports fans (Ostrowsky, 2016), who report higher levels of both positive and negative motivations for drinking than female fans (Pradhan et al., 2021). Triangulating this evidence with studies that show that alcohol is often combined with cocaine use (Ancrum et al., 2022), it is plausible to assume male sports fans are more likely to consume certain drugs. However, we must stress that there is no representative data yet to support this hypothesis.

As for other demographic patterns, the picture is less clear. To our knowledge, socioeconomic status (SES) has not been investigated or reported on in studies on individual-level drug consumption. If social class or SES were broadly attached to specific sports, then there is some empirical evidence (e.g., wastewater studies showing high levels of cocaine use at rugby games; Buckland, 2024) and much anecdotal evidence to suggest that drug use is evident across many types of sports and fan communities (e.g., horse racing; Rumsby, 2024 – Rugby; Prentice, 2024; darts; BBC, 2025), yet is likely under-reported outside of major events. We explore these demographic patterns in our data.

### Attitudes Toward Drug Use

The way in which fans respond to other fans consuming drugs is also important when investigating cultures of drug use. Previous research suggests that sports fans largely disapprove of physically aggressive or violent behavior committed by other fans (Donahue & Wann, 2009), although in some fan cultures (e.g., soccer) aggressive actions of organized fan groups (ultras, hooligans) are tolerated and even appreciated for their impact on stadium atmosphere and club reputation (e.g., Rookwood & Pearson, 2012). Taking this further, the perception that one's peers and/or wider groups do not condone such behavior is associated with a reduced willingness to behave in ways deemed ‘morally questionable’ at sports games (Sheehy & Maurice, 2022). In contrast, alcohol consumption, despite its association with fan disorder (though this link is not causal, see Ostrowsky, 2016), is widely tolerated in sports, evidenced by near omnipresent sponsorship deals and sales at events (Gallas, 2012). For instance, surveys among English and Scottish soccer fans indicate high levels of support for the consumption and availability of alcohol during and before matches, alongside a reluctance to attribute trouble in or around sports grounds to drinking (Purves et al., 2021).

In comparison, research that considers perceptions of drug use by other fans is distinctly lacking. This is a notable gap given that cocaine use is becoming increasingly normalized in leisure activities, including the electronic dance music club scene (Cristiano, 2024) and soccer fandom (Punzi, 2022), as well as more generally as an accompaniment to alcohol (Ancrum et al., 2022). Supporting these trends, a recent analysis of soccer chants in Greece found that references to drug use are becoming increasingly common, even serving as part of the self-identification process for Greek Ultras (Skliamis & Chatzinakos, 2023). Therefore, understanding not just what other fans think about drug use, but also their willingness to engage with or sanction this behavior, is vital to working with fan cultures and sports policymakers.

### Identity Fusion Theory

Identity fusion is a psychological construct that captures a visceral sense of oneness between the personal self and a group identity (Gómez et al., 2025; Swann et al., 2009, 2012). While it builds on foundational concepts from Social Identity Theory (SIT; Tajfel & Turner, 1979), identity fusion is both conceptually and empirically distinct from traditional social identification. Whereas SIT posits that individuals psychologically shift from personal to social identity depending on context (Turner et al., 1987), fusion theory proposes that personal and group identities can become functionally equivalent and simultaneously active, i.e., when they are *fused*. In this state, group membership is not merely an external affiliation but becomes internalized into the core self. This fusion of identities leads to a uniquely strong motivational force, enabling extreme pro-group behaviors, including acts of self-sacrifice or norm-defiance on behalf of the group as established among survivors of national disasters (Buhrmester et al., 2015), frontline soldiers (Whitehouse et al., 2014), and even soccer fans (Newson et al., 2016, 2023).

Over the past decade, identity fusion has been validated in diverse populations and settings, with empirical studies spanning over 100 countries (Tunçgenç et al., 2023). The construct has proven to be distinct from identification in both experimental and field settings (Gómez et al., 2011; Swann et al., 2009). Fusion predicts stronger behavioral outcomes than identification alone, particularly in contexts requiring high-cost commitment, intense loyalty, or norm-challenging behavior. For instance, Bortolini et al. (2018) found that fused soccer fans in Brazil were more likely to endorse extreme action in defense of their team. Similarly, White et al. (2021) demonstrated that fused Australian fans were more likely to endorse self-sacrifice, while identified fans were more likely to have outgroup prejudice without associated extreme behaviors. These findings highlight that fusion is not just a stronger form of identification but a qualitatively different mode of group alignment, characterized by an irrevocable psychological merging of the self with the group.

The relevance of identity fusion for sport has been increasingly recognized in recent years. Newson (2019) indicated that highly fused soccer fans in the UK often adopt ritualistic behaviors and report emotional intensity that transcends casual fandom, aligning with classic anthropological notions of communitas and collective effervescence (Turner, 1967). Moreover, fusion has been linked to aggressive or norm-breaking fan behaviors, such as drug use and violence, especially in highly charged matchday environments (Newson, 2021). The extreme affective bonds observed in fused fans appear to make them more susceptible to both elevated prosocial commitment and antisocial behavior, depending on the cultural context and perceived threat to the group (Newson et al., 2023). These dynamics underscore the explanatory power of fusion theory in understanding fan behaviors that deviate from societal norms yet remain highly consistent with group norms.

Despite a growing body of research in psychology, anthropology, and neuroscience (e.g., Swann et al., 2012; Whitehouse, 2018), the concept of identity fusion has rarely been explored in constructivist or sociological disciplines. While Newson's (2019) work applied fusion to cultural identity in sport in an article directed toward sports sociologists, few empirical studies have examined its sociological dimensions, particularly within the context of substance use. Yet fan cultures, especially in sport, are complex spaces where personal and collective identities converge, often under conditions of intense emotion, spectacle, and ritual. The strong foundations of understanding drug use at national or cultural levels within sociology (Becker, 1953; Measham, 2004; Pearson, 2001) provide an ideal platform from which to unite explorations of fan culture from psychological and sociological perspectives.

Investigating drug use across different national cultures and sporting subcultures, using both fusion and identification as distinct but interacting constructs, may help explain currently underexplored differences in attitudes and behaviors. This dual-level approach offers the potential to better understand why some fan groups or individuals engage in collective transgression while others do not, and how this is shaped by the intensity of their psychological bond to the team.

## Research Questions

As a first step to understanding widespread patterns of drug use across sports and continents, we examine the prevalence and motives of drugs use between fan groups in the UK and US, focusing on the most popular team sports by live attendance (UK; soccer, rugby, cricket. US; football, baseball, basketball. ice hockey; Two Circles, 2024, 2025). Although MLS soccer has highly similar attendance rates to ice hockey, the latter was prioritized as part of a separate project that contrasts fan conduct between high and low-contact US sports.

We explore drug culture by asking *what* drugs are used, *where* they are consumed, and *why*, by analyzing self-reported and observed use in surveys conducted with self-identified sports fans who attended at least one stadium event in the last 12 months. We then ask *who* consumes drugs and analyze the roles of fandom, team bonding (identification and fusion), and demographics such as age, gender, and SES to understand correlates of drug use. Finally, we ask *when* drug consumption is and is not tolerated*,* and explore fans’ willingness to sanction drug use with a focus on the role of collective identities, which typically inform social norms and efforts to police in-group transgressions.

## Method

Sample sizes were determined following a priori power analyses using G*Power (Faul et al., 2007) which indicated that a minimum sample of *N* = 322 fans per sports group was required to detect small effects (ω = .10) conducting chi-square analyses between a minimum of three groups at a power of .80 (error probability = .05). We aimed to oversample by ∼10% and recruit at least *N* = 360 fans per group (a total of 2,520 across sports and countries). Surveys were distributed via the crowdsourcing platform Prolific, an online participant recruitment service commonly used in academic research, using pre-screening tools to target UK and US nationals who regularly watch one of the target sports. Participants were reimbursed the equivalent of ∼£7.50 per hour for taking part. Ethical approval for this research was granted by the Research Ethics Committee of the School of Social Sciences at the University of Kent (ID: 1078).

### Sample

**Figure 1.**
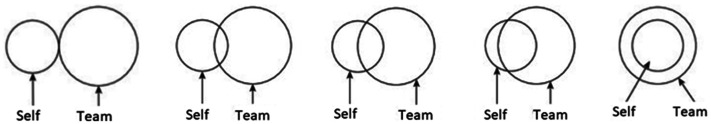
Pictorial Measure of Identity Fusion Adapted from Swann et al. (2009).

*Note:* Participants who selected the very right-hand image were coded as fused.

A total of *N* = 1,208 individuals took part in the UK survey. The eligible sample contained data from *N* = 1,085 sports fans (*M*_age_ = 43.34, *SD*_age_ = 13.34) after excluding participants who had not visited a stadium in the last 12 months (*n* = 20), did not provide consent (*n* = 1), did not complete the survey (*n* = 25), or completed the survey in under 1/3 of the median time (<85 s, *n* = 77). The majority self-identified as male (75.7%, female 23.8%, 0.4% non-binary, 0.2% prefer not to say), and 45.5% had attended a game in a stadium once or twice in the last 12 months (27.3% every other month, 19.9% once or twice a month, 7.3% almost every week). Over 80% indicated that they identified with their favorite sports team (scored above the midpoint on the team identification scale), and nearly 10% were fused to their team (see Figure 1). The sample consisted of 361 rugby fans, 361 soccer fans, and 363 cricket fans.

*N* = 1,757 individuals took part in the US survey, with a final eligible sample of *N* = 1,471 (*M*_age_ = 38.91, *SD*_age_ = 12.03). 286 responses were excluded, including those who did not provide consent (*n* = 4), indicated they had not visited a venue in the past 12 months (*n* = 245), withdrew from the survey (*n* = 35), or completed the survey too fast (*n* = 2). The majority self-identified as male (55.3%, female 43.4%, 0.6% non-binary, 0.3% Transgender, 0.3% prefer not to say). 51.3% attended a game in a stadium once or twice in the last 12 months (18.4% every other month, 20.1% once or twice a month, 10.3% almost every week (during the season). Over 83% indicated that they identified with their favorite sports team, and 12.6% were fused to their team. The sample contained 364 baseball, 376 basketball, 373 football, and 358 ice hockey fans. Full breakdowns of demographics and fan characteristics between groups can be found in Supplementary Materials (SM) Tables 1.1–1.2.

### Measures

Surveys were adjusted for British and American spelling but were identical unless specified otherwise. Complete scale descriptions can be found in section 2 of the supplementary materials.

Participants were asked how frequently they attended games in stadiums within the last 12 months (1 = “*never”* to 5 = “*almost every week”*), and to indicate their respective sports fandom (1 = “*not at all a fan”* to 5 = “*super fan”*). They were then asked to what extent they agreed with the statement: “I identify with my favorite [insert sport] team” (where 1 = “*strongly disagree”* to 5 = “*strongly agree”*, based on Postmes et al., 2013). Participants were then shown a diagram that depicted five pairs of circles adapted from Swann et al. (2009) (see Figure 1). Participants were informed that, within each pair, one of the circles represented themselves and the other circle represented their favorite sports team, with greater overlap representing a greater degree of perceived closeness. Participants were asked to select which of the pairs of circles best described their relationship with their team. Those who indicated a complete overlap between self and team were coded as fused (=1).

We measured general recreational drug use, and both recreational drug and alcohol use in or around sports venues in the last 12 months using binary items (1 = “*Yes”*, 0 = “*No”*). Those who indicated drug use at games completed relevant follow-up questions, including frequency of consumption at games (1 = “*once or twice”* to 4 = “*all the time”*), and which specific substance they had consumed at games. Participants could select all that apply from the following list: cocaine, cannabis, MDMA/ecstasy, ketamine, LSD/acid, Rohypnol, amphetamines, mephedrone, codeine, synthetic cannabinoids/spice, other -please specify. The UK survey also listed GHB. Users further responded to 10 items from Drazdowski et al.'s (2020) scale measuring motivations for substance use, covering various individual, social, and functional motives. The list of 10 items participants could choose from was: “To experiment – see what it's like”, “To increase the effects of some other drug (s) and alcohol”, “To fit in with a group I like.”, “because of boredom”, “To increase the effects of some other drug(s) and/or alcohol”, “To decrease (offset) the effects of some other drug(s) and/or alcohol”, “To be more alert/ready for a fight”, “Don’t know”, and “For a different reason”. The survey also included items to understand motivations to refrain from drug use at games, among those participants who said they used drugs but not in stadiums. They could select any of the following 5 items: “Risk of getting caught”, “It doesn’t fit with watching a game”, “I’m not with people who would tolerate it”, “Don't know”, and “Other”.

All participants indicated how often, over the last 12 months, they witnessed other fans under the influence of, or consuming recreational drugs in or around a relevant stadium (0 = *never* to 4 = *always*), which was recoded into a binary (1 = *at least once*, 0 = *never*). Those who selected ‘at least once’ were asked to select from a list of possible locations. These were: “in bathrooms”, “in the stands”, “on the transport before the game”, “on the streets”, “elsewhere”, and “don’t know”. Participants were also asked what types of drugs were consumed (identical to the respective list of possible drugs consumed). Participants also indicated how frequently they consumed alcohol when attending matches (1 = “*never”* to 5 = “*always”*).

Participants indicated their support for sanctions (aside from a possible criminal conviction) against fans who consume drugs at games (1 = “*no ban”* to 5 = “*a lifelong ban from all stadiums”*). In the UK survey, this was measured using two items which referred to the use of ‘class A drugs’ and ‘class B/C drugs’. In the US survey, because the country does not use the same drug classification system as the UK, this was measured using five items which referred to the use of specific substances, including ‘cocaine’, ‘cannabis’, and ‘Hallucinogens (e.g., MDMA/Ecstasy, LSD)’.

Lastly, the survey captured participants’ age (in years), their subjective SES on a scale from 1–10 (based on Adler et al., 1994, where 10 represents “the people who are best off – those who have the most money, the most education, and the best jobs”, and 1 represents “the people who are worst off”), and their gender (male, female, non-binary, transgender, prefer to self-describe, prefer not to say). Gender was recoded into a binary indicator (1 = *male*, 0 = *not male*). US participants also indicated whether they lived in a state where recreational cannabis use is legal (1 = “*Yes”*, 0 = “*No”*).

## Analyses

The prevalence of self-reported and observed drug consumption was examined using binary logistic regression analyses. Models predicting general drug use controlled for demographics (age, gender, and SES), and models predicting drug use at sports venues controlled for demographics and venue visit frequency and included fandom and team bonding (identification and fusion) as additional predictor variables. Models predicting drug observations controlled for demographics and own drug consumption, to account for familiarity with signs of drug consumption. Support of sanctions for drug use was tested using linear regression models including sports, demographics, self-reported and observed substance use, fandom, and team bonding. For a general overview, descriptive statistics of self-reported and observed substance use among all groups are reported in Table 1. Drug consumption statistics reported in the manuscript for US samples include responses from participants who live in states where cannabis consumption is legal. Group differences in locations of drug use, motivations to consume or refrain from consumption were tested with chi-square analyses, and adjusted standardized residuals (ASR) were used to further explore significant deviations from expected proportions.

**Table 1.** Prevalence of Recreational Drug Use at Sports Venues in the UK and US.

	Soccer	Rugby	Cricket	All UK	All US	Baseball	Basketball	Football	Ice Hockey
Sample *n*	361	361	363	1085	1471	364	376	373	358
Self-reported drug use									
% in general	17.2	17.7	7.7	14.2	37.0	34.3	40.2	37.3	36.3
% at a game	8.9	8.3	2.2	6.5	22.9	22.3	23.9	20.4	25.1
% Cannabis	6.1	5.3	1.4	4.1	19.6	20.1	19.4	17.4	21.5
% Cocaine	3.6	4.4	1.1	3.0	2.8	2.7	2.4	2.9	3.1
% MDMA/ Ecstasy	0.8	0.6	0	0.5	0.8	0.5	1.1	0.8	0.8
% Ketamine	0.8	0.6	0	0.5	0.7	0.5	1.3	0.3	0.8
% LSD/Acid	0.6	0.3	0	0.3	0.6	0.8	0	0.8	0.8
% Rohypnol	0	0	0	0	0.3	0.3	0.5	0	0.3
% Amphetamine	0	0.6	0	0.2	1.8	1.9	0.8	2.1	2.2
% Mephedrone	0	0.6	0	0.2	0.5	0.8	0.5	0	0.6
% Codeine	1.9	0.6	0.3	0.9	1.6	1.6	1.9	1.1	1.7
% Synthetic cannabinoids/Spice	0	0	0	0	1.2	1.1	1.1	1.1	1.7
% GHB	0	0	0	0	—	—	—	—	—
% Other	0	0.3	2.2	0.1	1.2	0.3	1.1	1.6	2.0
% Drank alcohol at least half the time	48.8	68.1	60.1	59.0	54.9	54.4	49.5	57.6	58.1
Observed drug use of other fans									
% at a game	75.9	60.4	46.6	60.9	76.6	76.4	76.6	81.2	72.1
% Cannabis	44.0	31.3	22.6	32.6	61.7	63.5	58.2	67.8	57.3
% Cocaine	39.1	37.1	19.0	31.7	19.2	16.8	18.1	19.8	22.1
% MDMA/ Ecstasy	6.6	3.3	1.1	3.7	4.6	4.4	4.5	4.6	5.0
% Ketamine	6.1	4.2	2.5	4.2	1.8	2.2	2.1	0.5	2.2
% LSD/Acid	1.7	0.3	1.1	1.0	2.4	3.0	1.4	1.9	3.6
% Rohypnol	0.6	0	0	0.2	0.5	0.3	0.8	0.5	0.6
% Amphetamine	3.6	3.3	0	2.3	5.7	3.6	6.9	7.2	5.0
% Mephedrone	0.6	0.6	0.3	0.5	1.8	2.7	2.1	1.3	0.8
% Codeine	2.8	1.4	0.3	1.5	5.6	5.8	8.0	3.8	4.7
% Synthetic cannabinoids/Spice	3.6	1.4	0.8	1.9	4.3	5.8	3.2	4.8	3.4
% GHB	0.6	0.6	0	0.4	—	—	—	—	—
% Other	0.3	0.3	0.6	0.4	0.7	0.8	0.8	0.8	0.6

*Note*. The percentages in this table relate to sample *n*s in the top row (i.e., all fans who took the survey) and are not equivalent to the percentage reported in text which reflect the choice of substance among drug users only.

## Results

### Drug Use Between Countries

The overall prevalence of recreational drug use within the last 12 months was significantly higher in the US samples compared to the UK, both in general (37% vs 14.2%) and at games (22.9% vs 6.5%). Models predicting drug use based on the data of all UK and US participants (n = 2556), controlling for demographic characteristics, showed that US fans were over 3 times more likely to have taken drugs overall (*OR *= 3.30, 95% CI: [2.68, 4.06], *p* < .001, χ^2^(4) = 230.48), and over 4 times higher at sports venues (*OR *= 3.98, 95% CI: [3.00, 5.27], *p* < .001, χ^2^(8) = 200.97) compared to British fans. In these models, fandom and team bonding were not associated with consumption at games (*p* values > .110). Americans were also more likely to have observed drug use at games (76.6% vs 60.9%), but the gap was less pronounced than for self-reported consumption (*OR *= 1.55, 95% CI: [1.29, 1.88], *p* < .001, χ^2^(7) = 279.16) (see SM Table 3.1 for full results). In contrast, the share of fans who indicated to have drunk alcohol at least half the time when they attended games was lower in the US (54.9%) than in the UK (59%) (*OR *= 0.83, 95% CI: [0.70, 0.98], *p* = .003, χ^2^(5) = 31.03).

### Drug Type

Among US sports fans who used drugs in and around venues, cannabis was by far the most common substance (85.5%), followed by cocaine (12.5%), amphetamines (7.7%), codeine (6.8%), and spice (5.3%). Correspondingly, the substances most commonly identified by those who observed others’ drug use were cannabis (80.2%) and cocaine (25%), while less frequently identified drugs included amphetamine (7.4%), MDMA (6%), codeine (7.3%), and spice (5.6%). Among UK-based fans who said they took drugs in the stadium, cannabis (64.3%) and cocaine (47.1%) were the most commonly used substances, while a variety of drugs were consumed only by smaller proportions of drug users (codeine = 14.3%; ketamine = 7.1%, MDMA = 7.1%). There were no associations between specific substances consumed and sports. Correspondingly, the most commonly identified substances consumed in and around stadia were cannabis (53.4%) and cocaine (52%). In contrast, other drugs were identified much less frequently (e.g., MDMA 6.1%, ketamine 7%, amphetamines 3.8%, spice 3.2%). Among fans who observed drug taking, cocaine was specifically identified by a higher proportion of rugby fans (60.3%) compared to cricket fans (40.8%) (χ^2^(2) = 16.31, *p* < .001), and by roughly half of soccer fans (48.5%) (see SM Table 3.5 for full results).

### Locations

Inside sports venues, drug use was observed most often in bathroom facilities, at a slightly higher rate in the UK compared to the US (χ^2^(1) = 5.84, *p* = .016), whereas more US fans had observed drug use in the stands (χ^2^(1) = 23.34, *p* < .001), which could reflect the higher prevalence of cannabis use in US samples and the higher acceptance of smoking in open spaces. Outside of venues, drugs were observed most often in the streets, at a higher rate in the US than in the UK (χ^2^(1) = 29.57, *p* < .001), while observations of drug use on transport before games were similar (χ^2^(1) = 0.66, *p* = .418) (see Table 2).

**Table 2.** Locations Where Fans Observed Drug Use When Attending Games.

Variable	Soccer	Rugby	Cricket	All UK	All US	Baseball	Basketball	Football	Ice Hockey
Sample *n*	274	219	169	662	958	240	249	252	217
% In bathrooms	56.9	57.1	46.2	54.2	48.3	47.5	51.9	45.7	48.3
% In the stands	32.5	34.4	27.2	31.8	43.3	47.5	37.2	47.9	40.4
% Transport before game	36.9	35.8	25.3	33.5	31.7	27.3	30.6	35.4	33.2
% In the street	55.5	43.8	47.3	49.5	62.7	62.2	66.8	58	64.1

*Note.* The percentages are based on subsamples of fans who reported observing drug use among other fans. Participants selected all that applied; the percentages do not add up to 100%.

There was relatively little variation between sports within countries. In the US, only observed drug use in the stands varied between fans (χ^2^(3) = 9.87, *p* = .020), with fewer such observations among basketball fans (*ASR* = −2.4, *p* = .016), and hockey fans (*ASR* = −1.1, *p* > .050). Similar to the country differences for drug observations in open spaces, a pattern of lower observed consumption in closed-roofed venues could reflect the popularity of cannabis among US sports fans. In the UK, differences were found in observed drug use on transport before the game (χ^2^(2) = 7.03, *p* = .030), which was less common among cricket fans (*ASR* = −2.6, *p* = .009), and in drug use observed in the streets (χ^2^(2) = 7.04, *p* = .030), which was more common among soccer fans (*ASR* = 2.6, *p* = .009) and less common among rugby fans (*ASR* = −2.1, *p* = .036) (see full results in SM Tables 3.6–3.7).

### Motivations

Similar motivations for drug use in sports settings emerged in both countries. The two most commonly cited reasons were to feel good and get high (US = 81.0%; UK = 61.4%) and have fun with friends (US = 71.2%; UK = 62.9%). Others indicated they took drugs to experiment (US = 13.9%; UK = 22.9%) or to fit in with another group (US = 11.6%; UK = 11.4%). There were also indicators of some functional drug use, either to increase (US = 11.0%; UK = 17.1%) or decrease (US = 3.0%; UK = 7.1%) the effects of other substances or alcohol, or to be alert for a fight (US = 5.3%; UK = 4.3%).

We further examined whether any motivations were uniquely associated with the most commonly reported substances (cocaine, cannabis). In the UK, cocaine was uniquely associated with the motive to decrease the effects of other drugs or alcohol (χ^2^(1) = 6.04, *p* = .014). In the US sample, cocaine was also associated with functional motives, to increase (χ^2^(1) = 15.98, *p* < .001) or decrease (χ^2^(1) = 7.47, *p* = .006) the effects of other substances, or to be more alert for a fight (χ^2^(1) = 7.97, *p* = .005). Moreover, it was linked with motives to experiment (χ2(1) = 9.13, *p* = .003) and to fit in with a group (χ^2^(1) = 14.28, *p* < .001). In contrast, cannabis use was significantly linked to the motivation to have a good time with friends (χ^2^(1) = 4.05, *p* = .045) (see SM Tables 3.8 & 3.10 for full results). Among fans who used drugs in the last 12 months but not at sports venues, the reasons for refraining were also similar in both countries. The most common reason was that drug use did not fit with watching a game (US = 67.3%; UK = 89.3%), while others were concerned by the risk of getting caught (US = 30.8%; UK = 36.3%) and the disapproval of the people they attend games with (US = 21.2%; UK = 33.3%). Among “other reasons” cited by fans (US = 15.4%, UK = 13.1%), there was respect for other fans, the need to stay sober for driving to and from the venue, and avoidance of side effects. There were no differences between fan groups within countries (see SM Tables 3.7 & 3.11).

### Correlates of Drug Use

In the US sample, there were no significant differences in drug use between sports fans, for consumption overall (*p* values > .449), at games (*p* values > .227), or of specific substances (*p* values > .180). There was some variation for observed consumption as football fans were almost twice as likely to observe drug use in or around stadia at least once (*OR *= 1.90, 95% CI: [1.32, 2.72], *p* < .001, χ^2^(9) = 116.02), and observe it more frequently, compared to ice hockey fans (see SM Tables 3.2 & 3.4 for full model results).

Consumption at games in the US was not associated with sports fandom or team bonding (identification and fusion) (*p* values > .541), but there were significant links with attendance frequency (*OR *= 1.27, 95% CI: [1.21, 1.43], *p* < .001) and socioeconomic status (*OR *= 0.92, 95% CI: [0.86, 0.99]), *p* = .040, χ^2^(10) = 30.07), such that more frequent game attendance and lower placement on a socioeconomic ladder were both associated with self-reported consumption. In the UK, there was significant variation with regard to both self-reported consumption and observations in and around stadiums. Rugby fans were over three times, and soccer fans two times more likely to have consumed drugs at games than cricket fans (rugby *OR *= 3.57, *p* < .001, soccer *OR *= 2.32, *p* = .009). Team bonding (identity fusion) was also significantly linked to drug consumption, as UK fans who were fused to their favorite team were twice as likely to admit to drug use at games (*OR *= 2.31, 95% CI: [1.15 to 4.66], *p* = .019). Similar to the US, attendance frequency (*OR *= 1.47, 95% CI: [1.10, 1.98], *p* = .010) and SES (*OR *= 0.77, 95% CI: [0.65, 0.91], *p* = .002) showed significant associations, as well as age (*OR *= 0.96, 95% CI: [0.94, 0.98], *p* < .001) χ^2^(9) = 69.43, Nagelkerke *R*^2^ = .16, *p* < .001). Moreover, group differences were also evident for observations of drug consumption. Soccer fans were almost three times more likely to have witnessed drugs used in or around stadia (*OR *= 2.84, 95% CI: [2.01, 4.00]) compared to cricket fans and two times more likely than rugby fans (*OR *= 1.97, 95% CI: [1.39, 2.80]). Rugby fans were also about 1.5 times more likely to observe drug use than cricket fans (witnessed drug use; *OR *= 1.44, 95% CI: [1.06, 1.96]) (see SM Table 3.8 for full results).

### Support for Drug Sanctions

Among US fans, football fans showed stronger support of sanctions for cocaine use at games (*B* = 0.24, *SE* = 0.10, 95% CI: [0.04, 0.43], *p* *=* *.018*) and Basketball fans of sanctions for cannabis use (*B* = 0.22, *SE* = 0.09, 95% CI: [0.05, 0.39], *p* *=* *.012*), but otherwise there were no sport effects. Those who had consumed drugs in general were consistently more opposed to sanctions (*B*s = −0.28 to −0.50, 95%CIs [−0.69, −0.10]; *p* values < .003), whereas those who attended more frequently were consistently more in favor (*B*s = 0.08 to 0.18, 95%CIs [0.01, 0.25]; *p* values < .023). Collective sports identities showed divergent effects. Stronger identification with one's favorite team was associated with leniency for consumption of any drug (*B*s = −0.10 to −0.17, 95%CIs [−0.28, −0.01]; *p* values < .048), whereas fused US Sports showed support for harsher sanctions for the use of cocaine, cannabis and hallucinogens (*B*s = 0.39 to 0.48, 95%CIs [0.16, 0.70]; *p* values < .001). Fandom was not associated with sanctions (*p* values > .276) (see SM Table 3.13 for full results).

In the UK samples, there were no differences between sports regarding support of sanctions for either Class A or Class B/C drugs. For comparison with the US sample, Class A drugs include cocaine and hallucinogens, whereas cannabis is classified as a Class B drug (GOV.UK, n.d.). The UK classification system is based on the perceived harmfulness of the drug and informs sentencing recommendations, with the most severe sentences reserved for offenses involving Class A substances (Duddy & Downs, 2024). As in the US, fans who generally consumed drugs were significantly more lenient (*B*s = −0.72 to −0.79, 95%CIs [−1.03, −0.48]; *p* values < .001), but there were no effects of attendance (*p* values > .085) or other demographics. In contrast to the US, stronger team identification was associated with support of sanctions for use of both Class A drugs (*B* = 0.13, *SE* = 0.05, 95% CI: [0.02, 0.22], *p* = .016) and Class B/C drugs (*B* = 0.12, *SE* = 0.05, 95% CI: [0.02, 0.22], *p* *=* *.025*), while team fusion was also linked with harsher punishment for Class B/C drugs (*B* = 0.25, *SE* = 0.12, 95% CI: [0.01, 0.49], *p* = .044) (see SM Table 3.12 for full results).

## Discussion

Our findings confirm that recreational drug use is a common feature among fans at sports events in both the US and UK, supporting and extending previous research (Lemas et al., 2021; Montgomery et al., 2021; Newson, 2021). Among fans of the most popular spectator team sports who attended games in the past 12 months, the majority reported witnessing drug use in and around stadiums, while self-reported consumption levels were consistent with, or exceeded, national averages. In the US, 2 in 10 fans admitted to using drugs at games at least once in the past year, with nearly 8 in 10 observing others under the influence of substances other than alcohol. In the UK, self-reported drug use was lower, with fewer than 1 in 10 fans reporting consumption at matches, yet 6 in 10 had witnessed drug use among fellow spectators. Cannabis and cocaine emerged as the most frequently used substances in both countries, though cannabis use was notably higher in the US, where its legal status in half of all states likely contributes to its widespread presence at sporting events^1^.

There were notable differences in drug use patterns across different sports fan groups in the UK. Drug use was considerably more common among soccer and rugby fans, compared to cricket fans. Within these high-use groups, the prevalence of cocaine exceeded national averages (2.1% of adults in the UK; ONS, 2024, vs rugby 4.4%, soccer 3.6%), whereas cannabis use was slightly lower (6.8% of adults in UK, vs soccer 6.1%, rugby 5.3%), suggesting that drug consumption among sports fans does not simply mirror general population trends but is instead shaped by subcultural norms and event-specific contexts. In contrast, US sports fans were more homogenous in their drug use, perhaps reflecting a more commercialized or standardized sporting culture. Like the UK, the prevalence of cocaine use was higher, and cannabis use was lower than the national average (in the US, 2.4% of adults use cocaine vs 2.8% of sports fans, 26.6% use cannabis vs 19.2% of sports fans, UNODC, 2023). Greater drug use over alcohol use in the US, compared to the UK, may relate to socio-historic patterns, including a history of prohibition and fans relying on driving to attend games due to limited public transport and therefore not consuming alcohol.

Regarding the locations of consumption, our data relies on fan observations, rather than self-reports. The data suggests a logical pattern: Drugs are most commonly consumed in places that provide some privacy, either secluded in bathrooms or in relative anonymity on the streets, where behaviors like smoking are commonplace. Overall, these findings highlight that efforts to address drug use at games would require collaboration between public stakeholders and sports organizations, i.e., those who are responsible for venues. Given the prevalence of drugs in communal areas frequently used by families, like venue bathrooms, we encourage clubs to engage with their fan communities. By consulting fans, clubs can gain insights into their awareness and opinions on the issue, and work toward co-designing interventions that are both inclusive and age-appropriate.

Most fans indicated recreational motives to consume drugs, specifically to have fun and have a good time with friends. This is in line with the characterization of a carnivalesque fan culture, where the role of alcohol consumption is recognized for its cultural and social significance for cohesion among soccer fans (Bandura et al., 2024; Pearson, 2012). Our findings could suggest that drugs other than alcohol could also become a mainstay in British and American fan culture, if they are not already, which provides new evidence for patterns of the carnivalesque being non-alcohol specific. While our insights on whether motives are linked to distinct substances draw on relatively small subsamples of cocaine and cannabis users, the data suggested that cocaine use has a functional component. Functional cocaine use could reflect an alternative soccer carnivalesque, prolonging euphoria at games, and reducing side-effects of alcohol (Bandura et al., 2024). However, the broader implications of functional cocaine use, both on sports culture and fan behavior (e.g., see Newson, 2021), require further exploration.

Among the potential correlates of drug use, we observed some demographic trends. For instance, younger fans were more likely to consume drugs at games, aligning with established research indicating that youth is strongly associated with risk-taking (Steinberg et al., 2008). This finding is particularly relevant in the context of sports events, where drug use remains both illegal and socially non-normative. The link between SES and drug use, however, is more complex as different drugs have differing prevalence (e.g., cocaine is associated with both low and high incomes in the UK, ONS, 2024), and there is no clear evidence in the literature that links SES to drug use among fans. We did not observe gender effects, even though past studies have indicated that cocaine use and alcohol use tend to be higher among male fans (Newson, 2021; Ostrowsky, 2016), which might be a reflection of the small sub-sample of drug users in our study. Beyond individual demographics, fan characteristics and engagement levels also played a significant role in shaping drug use patterns. Frequent game attendees were more likely to indicate they consumed drugs, possibly reflecting increased familiarity with stadium environments and greater confidence in consuming drugs without fear of repercussions. However, as attendance frequency also correlated with observed drug use, it might also reflect increased frequency of exposure to drug-taking opportunities and others’ consumption.

In the UK, team bonding, especially in the form of identity fusion, was significantly associated with drug use at sporting events. This may align with a carnivalesque interpretation of fandom, wherein highly bonded fans seek emotional catharsis and solidarity through norm-breaking practices such as drug consumption. Fusion, in this context, appears to facilitate transgressive behaviors as a shared ritual that reinforces group identity, blurs boundaries between the personal and collective self, and contributes to a heightened sense of communitas (Páez & Rimé, 2014; Whitehouse & Lanman, 2014; Zabala et al., 2024). The data suggest that such drug use may not merely indicate individual hedonism but a form of embodied group expression, echoing earlier ethnographic work that positions soccer fandom as a site for ritual inversion and temporary release from social norms (Millward, 2006), i.e., the carnivalesque (Bakhtin, [1968] 2020; Giulianotti, 1995; Pearson, 2012).

In contrast, fan bonding in the US, whether through identification or fusion, was not associated with drug-taking behavior. This suggests that drug use in American sports contexts is less embedded in collective fan identity and more likely to be experienced as an individual act. Such a divergence may reflect broader cultural differences in the role of fan communities and the moral regulation of public space. In the US, where the commodification and individualization of fandom is arguably more pronounced, drug use may exist outside the boundaries of collective rituals and therefore escape the symbolic weight it holds in UK settings. These findings underscore the culturally contingent nature of identity fusion: while fusion may amplify group-based transgressions in one setting, it may serve as a regulatory or even conservative force in another. This speaks to the complex ways in which collective identities shape not only fan behavior but also attitudes toward acceptable expressions of belonging and resistance within sporting cultures.

Furthermore, identity clearly shaped attitudes toward sanctions, with striking cross-national differences. In the UK, stronger identification predicted support for harsher punishment of drug use, including Class A and B/C substances, while fusion was linked specifically to support for punishing B/C drug violations. This pattern suggests that norm-breaking can coexist with internal group regulation; fans may participate in transgression during games but still endorse sanctions to protect the group's boundaries and image afterwards, although we acknowledge that such patterns might be different among fan groups with distinct anti-authoritative histories (e.g., ultras, hooligans). In the US, the pattern was reversed: identification predicted leniency, possibly reflecting an inclusive stance toward fellow fans, while fusion predicted harsher punishment, indicating a desire to police in-group behavior and uphold internal norms. These findings highlight that identity processes are culturally contingent and possibly reflect broader national and cultural dynamics.

Viewed through the lens of the carnivalesque, this tension between sanctioned transgression and ‘moral’ (or at least legal) reassertion mirrors the cyclical structure of festival and release (Bakhtin, [1968] 2020), while identity fusion theory helps explain why fused individuals may act as both participants in and guardians of group norms, depending on cultural or subcultural context. Importantly, group fusion should not be understood as leading to single, uniform behavioral outcomes. Highly fused fans are intensely committed to their group, and as such, their actions are contingent on what they perceive to best serve the group's interests. This can result in norm-breaking behaviors where transgression is viewed as affirming identity and solidarity (e.g., carnivalesque expressions which may include disorder or drug taking), but also in strong support for sanctions where deviance is seen to threaten the group's reputation or cohesion. In both cases, the intensity of fusion may drive strong behavioral responses, though the direction of these responses is context-dependent (Newson et al., 2023).

In the US, higher overall drug consumption and the widespread view that the War on Drugs has failed may have shaped more lenient norms within fan communities, especially among those who identify with the group but are less intensely bonded. However, highly fused fans may resist this permissiveness, seeking to uphold a more ‘pure’ or law-abiding version of their team identity. In contrast, in the UK, drug use remains socially disapproved of and is harshly sanctioned in sports contexts (particularly soccer; UK Home Office, 2024), despite high national usage rates. Sports clubs often position themselves as inclusive, charitable institutions and pillars of the community, reinforcing a strong moral image. At the same time, matchdays can provide a carnivalesque escape from these pressures, allowing transgressive behaviors within a controlled space. Thus, while fusion in the UK may facilitate participation in this collective transgression, it may also be accompanied by support for post-hoc punishment as a means of maintaining the group's public image and internal boundaries. Understanding these dynamics is crucial for developing context-sensitive harm reduction and policy responses in sports environments.

## Limitations

​This study has several limitations that should be considered when interpreting the findings. First, we rely entirely on self-report measures in an anonymous online survey, and future work on this issue should verify findings with fieldwork in and around matchday events. Second, the use of non-representative samples limits the generalizability of our results to the broader population of sports fans. While recruitment via Prolific is known for high data quality (Douglas et al., 2023; Eyal et al., 2021) and participants are unlikely to be less honest in this platform compared to any other anonymized survey platform, it is also unlikely to include hard to reach fan subgroups (e.g., ultras, hooligans) that are commonly accessed by ethnographic research approaches, and whose attitudes and behaviors could plausibly deviate from those captured here. Future studies should at least account for membership in organized fan groups to potentially control for this factor. As such, our research should be triangulated with further ethnography building on existing contributions in the field of soccer fandom (e.g., Armstrong, 1998; Armstrong & Rosbrook-Thompson, 2015; Pearson, 2012; Rookwood & Pearson, 2012).

Third, the relatively small subsamples of drug users within our study may have led to underpowered analyses, potentially obscuring significant associations. Examining a broad spectrum of substances may have also reduced the specificity needed to draw precise conclusions on particular drugs. Reported witnessing of others’ drug use poses a further problem in that fans cannot be sure what drugs others are taking, beyond simple cues like smell for cannabis or nasal insufflation for cocaine, though other drugs, such as ketamine in the UK context, could easily be mistaken for cocaine. Lastly, the lack of data on venue types, competition levels or the ratio of home- vs away-game attendance (which historically differs between sports and fan cultures) prevents us from accounting for the impact of existing mechanisms to deter drug use at games, such as levels of security or policing, or speak to subcultural nuances linked to travelling fans (Stott et al., 2001) or supporters of different sports formats (e.g., rugby union vs rugby league). Future research should incorporate more representative sampling methods, larger drug-using subsamples, and focus on particular substances, which might vary based on geographical availability, to enhance the applicability of the findings to public health interventions and policy development, ideally with the consultation of fans themselves.​ We hope that larger-scale surveys will follow this research, triangulated with more nuanced methodologies such as ethnography.

## Implications and Conclusion

These findings also have important policy implications. In the UK, given the distinct differences between fan groups, targeted interventions tailored to specific sports and their fan cultures may be more effective than broad, one-size-fits-all policies. Soccer and rugby events, for instance, may require more considered harm reduction efforts, focusing on cocaine use, compared to cricket. A more generalized approach could be sufficient in the US, where fan behaviors appear more uniform. The contrast between these patterns and general population data further underscores the need for sport-specific policies. Our findings suggest that sports fans in both countries exhibit distinct consumption patterns that cannot simply be extrapolated from national drug use rates. Tailoring harm reduction and enforcement strategies to sports contexts is therefore essential for effective policy development and could be a springboard to provide education in an accessible environment that does not feel authoritative or patronizing. We do not advocate for harsher penalties, which could be counter-productive and miss an opportunity to provide education and safer attitudes toward drug taking.

Sport settings can be an effective messaging context, e.g., for health awareness (Curran et al., 2014; Elsey et al., 2024), but it is currently unclear whether, and to what extent, professional sports organizations are ready or willing to invest in harm reduction efforts. A first step for sports organizations could involve engaging with fan communities to better understand the prevalence and nature of drug use in different contexts, such as specific regions or competitions. Without knowledge about the scope of the issue and awareness of potential consequences of regulatory steps, stakeholders risk under- or overestimating the impact of policies to reduce drug consumption at games. This approach presents a low-risk opportunity for organizations to gain insights into how and why certain segments of their fanbase consume drugs at games, as well as the potential impacts on other stadium visitors.

These results provide insights into the prevalence of recreational drug use among sports fans in the US and UK, highlighting key demographic, behavioral, and sport-specific differences. While drug use at sporting events is a widespread phenomenon, its manifestations vary across contexts, necessitating tailored harm reduction strategies and policy responses. How might future research disentangle the complex role of identity and national culture in shaping fan drug use? This issue exists within a broader context where drug use remains illegal on a global scale, with substances often obtained through unregulated markets that pose significant risks, including uncertain potency, contamination, and the potential for serious adverse health effects. Further research is needed to directly inform interventions that promote safer environments, such as free on-site drug testing, while acknowledging the complex legal, health, and ethical dimensions of drug use at sporting events. Addressing these challenges through targeted education and harm reduction strategies will be crucial in ensuring both public well-being and the integrity of sporting events for the future.

## Supplemental Material

sj-docx-1-jss-10.1177_01937235251387699 - Supplemental material for Recreational Drug use at Sports Events in the US and UK Supplemental material, sj-docx-1-jss-10.1177_01937235251387699 for Recreational Drug use at Sports Events in the US and UK by Martha Newson, Linus Peitz and Mollie Ruler in Journal of Sport and Social Issues

## Notes

1. We tested how sensitive our findings are to our US sample's background from states where consumption of cannabis is legal. First, we replicated the analyses predicting drug use excluding participants from legal states, and a second time, we recoded the dependent variable to *not* consider individuals who reside in legal states and who report exclusive cannabis consumption as drug users (i.e., effectively predicting illicit drug consumption only). Both approaches yielded highly similar results to the models including legal cannabis users, showing significant higher likelihood of consumption by US participants, and no group differences between US spectator groups (see section 4 in the supplementary materials for model results).
