# Supplementary material for: Recreational Drug use at Sports Events in the US and UK
Source: J Sport Soc Issues. 2025 Oct 21;50(2):115–44. doi: 10.1177/01937235251387699 (PMC12919766; doi:10.1177/01937235251387699)
Supplement: sj-docx-1-jss-10.1177_01937235251387699 - Supplemental material for Recreational Drug use at Sports Events in the US and UK [file sj-docx-1-jss-10.1177_01937235251387699.docx]

Contents of Supplementary Materials for “A cross-sectional investigation into fan drug use at sports events in the US and UK: what, who, where, and why?”

[1. Sample Demographics and Fan Characteristics Between Spectator Groups 1](#_Toc195106427)

[2. Table of Measures 3](#_Toc195106428)

[3. Full Model Results for Manuscript Analyses 8](#_Toc195106429)

[4. Additional Analyses Considering Legal Cannabis Status in US States 20](#_Toc195106430)

# Sample Demographics and Fan Characteristics Between Spectator Groups

| Table 1.1  Demographics and Characteristics of Participant Groups from the UK | | | | | | |
| --- | --- | --- | --- | --- | --- | --- |
|  | Total  (N = 1085) | Soccer (n =361) | Rugby (n=361) | Cricket (n=363) |  |  |
| Variable | *M* (SD)/ % | | | | *Group differences* | Post-hoc/ASR |
| Age | 43.34 (13.34) | 41.73 (12.91) | 43.32 (13.17) | 44.96 (13.75) | *F*(2, 1084) = 5.33, *p* = .005, η^2^ = .01, | Soccer < Cricket (*p* =.003) |
| % Male | 75.67 | 77.01 | 71.19 | 78.79 | *χ^2^(2) =* 6.20, *p* =.045 | Rugby *ASR* = -2.4 (*p* = .016) |
| Socioeconomic status | 6.09 (1.38) | 6.01 (1.35) | 6.10 (1.42) | 6.16 (1.37) | *F*(2, 1084) = 1.05, *p* = .349, η^2^ = .002 |  |
| Frequency of game attendance | 2.89 (0.97) | 3.26 (1.05) | 2.80 (0.90) | 2.61 (0.83) | *F*(2, 714.70) = 43.08, *p* <.001, η^2^ = .08 | Soccer > Rugby (*p* <.001)  Soccer > Cricket (*p* <.001)  Rugby > Cricket (*p* = .007) |
| Fandom | 3.60 (0.72) | 3.94 (0.73) | 3.42 (0.67) | 3.44 (0.64) | *F*(2, 1084) = 68.36, *p* <.001, η^2^ = .11 | Soccer > Rugby (*p* <.001)  Soccer > Cricket (*p* <.001) |
| Team identification | 4.00 (0.75) | 4.29 (0.73) | 3.86 (0.69) | 3.85 (0.75) | *F*(2, 1082) = 43.67, *p* <.001, η^2^ = .08 | Soccer > Rugby (*p* <.001)  Soccer > Cricket (*p* <.001) |
| % Team fusion | 9.8 | 16.9 | 7.5 | 5 | *χ^2^(2) =* 32.49, *p* <.001 | Soccer *ASR* = 5.6 (*p* <.001)  Cricket *ASR* = -3.8 (*p* <.001) |
| Note. Socioeconomic status 0 – 10, Frequency of game attendance 1 – 6, Fandom 1 – 5, Team identification 1 – 5, Team fusion 1 = Yes. For the frequency of game attendance, we report the Welch *statistic* and Games-Howell post hoc tests due to a violation of the homogeneity of variances assumption (Levene *F=*(2,1082) = 14.47, *p* <.001). | | | | | | |

| **Table 1.2**  Demographics and Characteristics of Participant Groups from the US | | | | | | | |
| --- | --- | --- | --- | --- | --- | --- | --- |
|  | Total  (N = 1471) | Baseball (n =364) | Basketball (n=376) | American Football (n=373) | Ice Hockey (n=358) |  |  |
| Variable | M (SD)/ % | | | | | Group differences | Post-hoc/ASR |
| Age | 38.91 (12.03) | 40.61 (12.34) | 36.19 (10.69) | 39.98 (12.78) | 38.93 (11.77) | *F*(3, 812.04) = 11.07, *p* < .001, η^2^ = .02, | Basketball < Baseball/ American Football (*p* <.001)  Basketball < Ice Hockey (*p* =.002) |
| % Male | 55.3 | 65.4 | 64.1 | 43.2 | 48.6 | *χ^2^(3) =* 55.47, *p* <.001 | All groups (ASRs > 2.8, *p* values <.001) |
| Socioeconomic status | 5.61 (1.61) | 5.76 (1.62) | 5.62 (1.55) | 5.48 (1.64) | 5.56 (1.63) | *F*(3, 1467) = 1.99, *p* = .114, η^2^ = .002 |  |
| Attendance frequency | 2.89 (1.06) | 2.98 (1.02) | 3.01 (1.13) | 2.70 (1.01) | 2.89 (1.03) | *F*(3, 814.42) = 6.41, *p* <.001, η^2^ = .01 | American Football < Baseball (*p = .002*)  American Football < Basketball (*p* <.001) |
| Fandom | 3.72 (0.77) | 3.67 (0.73) | 3.82 (0.80) | 3.80 (0.80) | 3.58 (0.73) | *F*(3, 1467) = 8.29, *p* <.001, η^2^ = .02 | Baseball < Basketball (*p*  = .028)  Ice Hockey < Basketball/ American Football (*p* <.001) |
| Team identification | 4.02 (0.73) | 4.04 (0.68) | 4.06 (0.71) | 4.00 (0.79) | 3.96 (0.73) | *F*(3, 1467) = 1.44, *p* = .231, η^2^ = .003 |  |
| % Team fusion | 12.6 | 14 | 17 | 11.3 | 7.8 | *χ^2^(3) =* 15.39, *p* = .002 | Basketball *ASR* = 3.0 (*p* =.003)  Ice Hockey *ASR* = 9.61 (*p* = .002) |
| Note. Socioeconomic status 0 – 10, Frequency of game attendance 1 – 6, Fandom 1 – 5, Team identification 1 – 5, Team fusion 1 = Yes. For Age and Frequency of game attendance, we report Welch *statistics* and Games-Howell post hoc tests due to violations of the homogeneity of variances assumption (Age, Levene *F*(3,1467) = 5.16, *p =*.002; Game attendance, Levene *F*(3, 1467) = 4.74, *p* = .003). | | | | | | | |

# Table of Measures

| **Table 2**  Overview of Measures, Items, Scales and Sources | | | | |
| --- | --- | --- | --- | --- |
| Measure name | Items | Scale | Recoded (if applicable) | Source (if applicable) |
| Age | Please indicate your age in years |  |  |  |
| Subjective Socioeconomic Status | Think of the ladder below as representing where people stand in society. At the top of the ladder are the people who are best off – those who have the most money, the most education, and the best jobs. At the bottom are the people who are worst off – where would you place yourself on the ladder? | 1-10 |  | Adler et al. (1994) |
| Gender | How do you describe yourself? | male, female, non-binary, transgender, prefer to self-describe, prefer not to say. | 1 = male, 0 = not male |  |
| Cannabis legality (US only) | Do you live in a US state where recreational cannabis use is legal? | Yes/No |  |  |
| Game attendance | Over the past 12 months, how often have you watched a [insert sport] game live in a [stadium, arena, ballpark] in the UK (study1)//US (study 2)?” | 1 = never, 2 = once or twice, 3 = every other month, 4 = once or twice a month, 5 = almost every week. |  |  |
| Sports fandom | How much of a [insert sport] fan do you consider yourself?” | 1 = not a fan at all, 2 = occasional fan; I follow it occasionally, but it's not a major interest, 3 = moderate fan; I enjoy watching games and follow some news, but I’m not super dedicated; 4 = dedicated fan; I follow teams/players closely and I rarely miss a game, 5 = super fan; [insert sport] is a big part of my life. I live and breathe it. |  |  |
| Team identification | I identify with my favourite [insert sport] team.” | 1 = strongly disagree to, 2 = disagree, 3 = neither agree nor disagree, 4 = agree, 5 = strongly agree. |  | Postmes et al. (2013) |
| Team fusion | In the below pictures, you will see two circles. One of the circles represents you, and the other circle represents your favourite [insert sports] team. The overlap between the circles represents the degree of closeness between yourself and your team. Please select which picture best describes the relationship between you and your team. | A = 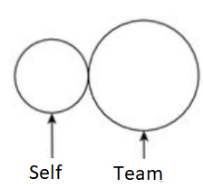  B = 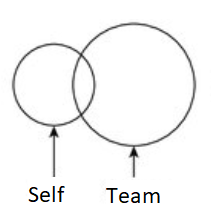  C =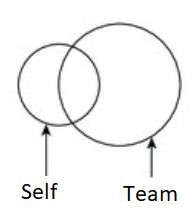  D = 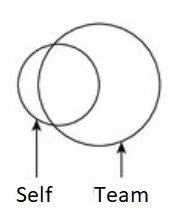  E = 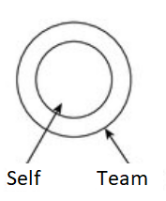 | 1 = fused (option E), 0 = not fused | Swan et al. (2009) |
| Alcohol consumption | Over the last 12 months, how often did you drink alcohol when watching a game in a stadium? | 1 = Never, 2 = Sometimes, 3 = About half the time, 4 = most of the time, 5 = always. |  |  |
| Drug consumption | Over the last 12 months, have you consumed any recreational drugs at all, including non-medical use of prescription drugs? | 1 = Yes, 0 = No |  |  |
| [if drug consumption = Yes] Drug consumption at games | Over the last 12 months, did you consume/were you under the influence of any recreational drugs when visiting a [insert sport] stadium?” | 1 = Yes, 0 = No |  |  |
| Drug consumption substance type | What kind of drugs did you consume in the stadium? Please select all that apply.” | Cocaine, Cannabis, MDMA/Ecstasy, Ketamine, GHB (not included for US survey), LSD/Acid, Rohypnol, Amphetamines, Mephedrone, Codeine, Synthetic cannabinoids/Spice, Other -please specify. |  |  |
| [if drug consumption at games = Yes]  Motivations to use drugs | What was your motivation to consume drugs while watching a game? Please select all that apply. | To experiment – to see what it’s like; to feel good or get high; to have a good time with my friends; to fit in with a group I like; because of boredom; To increase the effects of some other drug(s) and/or alcohol; To decrease (offset) the effects of some other drug(s) and/or alcohol; To be more alert/ready for a fight; Don’t know; For a different reason (please specify). |  | Based on Drazdowski et al. (2020) |
| if drug consumption at games = No]  Motivations to **not** use drugs | You indicated that you have consumed drugs, but not in a stadium. What stopped you from consuming drugs in a stadium? Please select all that apply.” | Risk of getting caught; It doesn’t fit with watching a game; I’m not with people who would tolerate it; Don't know; Other reasons -please specify. |  |  |
| Observed drug consumption | Over the last 12 months, how often have you witnessed other fans under the influence of/consuming recreational drugs in or around a football stadium?” | 1 = Never, 2 = Once or twice, 3 = several times, 4 = regularly, 5 = always. | 1 = at least once, 0 = never |  |
| [if observed drug consumption >1]  Observed drug consumption location | Did you witness where these recreational drugs were consumed? If so, please select all that apply.” | in bathrooms, in the stands, on the transport before the game, on the streets, elsewhere, don’t know. |  |  |
| [if observed drug consumption >1]  Observed drug consumption type | Did you witness what kind of drugs were consumed? If so, please select all that apply.” | Cocaine, Cannabis, MDMA/Ecstasy, Ketamine, GHB (not included for US survey), LSD/Acid, Rohypnol, Amphetamines, Mephedrone, Codeine, Synthetic cannabinoids/Spice, Other -please specify. |  |  |
| *Note. British s*pelling was presented in UK survey. | | | | |
| References  Adler, N. E., Boyce, T., Chesney, M. A., Cohen, S., Folkman, S., Kahn, R. L., & Syme, S. L. (1994). Socioeconomic status and health: The challenge of the gradient. *American psychologist*, *49*(1), 15-24.  Drazdowski, T. K., Kelly, L. M., & Kliewer, W. L. (2020). Motivations for the nonmedical use of prescription drugs in a longitudinal national sample of young adults. *Journal of Substance Abuse Treatment*, 114, 108013.  Postmes, T., Haslam, S. A., & Jans, L. (2013). A single‐item measure of social identification: Reliability, validity, and utility. *British journal of social psychology*, *52*(4), 597-617.  Swann Jr, W. B., Gómez, A., Seyle, D. C., Morales, J., & Huici, C. (2009). Identity fusion: the interplay of personal and social identities in extreme group behavior. *Journal of personality and social psychology*, *96*(5), 995. | | | | |

# Full Model Results for Manuscript Analyses

| **Table 3.1**  Results of Logistic Binary Regression Models Predicting Self-Reported Drug Use Anywhere (Model 1), Self-Reported Drug Use at Games (Model 2), and Observed Drug Use at games (Model 3) Based on Country and Demographic Characteristics | | | | | | | | | | | | | | | | | | |
| --- | --- | --- | --- | --- | --- | --- | --- | --- | --- | --- | --- | --- | --- | --- | --- | --- | --- | --- |
| Parameter | Model 1: Consumed drugs anywhere | | | | | | Model 2: Consumed drugs at games | | | | | | Model 3: Observed drugs at game | | | | | |
|  |  |  |  |  | 95% OR CI | |  |  |  |  | 95% OR CI | |  |  |  |  |  |  |
| Parameter | B | SE | *p* | OR | Lower | Upper | B | SE | *p* | OR | Lower | Upper | B | SE | *p* | OR | Lower | Upper |
| Country | 1.19 | 0.11 | <.001 | 3.30 | 2.68 | 4.06 | 1.38 | 0.14 | <.001 | 3.98 | 3.00 | 5.27 | 0.44 | 0.10 | <.001 | 1.55 | 1.29 | 1.88 |
| Age | -0.02 | 0.00 | <.001 | 0.98 | 0.97 | 0.99 | -0.01 | 0.00 | .002 | 0.99 | 0.98 | 0.99 | -0.02 | 0.00 | <.001 | 0.98 | 0.98 | 0.99 |
| Gender | 0.25 | 0.10 | .012 | 1.28 | 1.05 | 1.55 | 0.16 | 0.12 | .172 | 1.18 | 0.93 | 1.49 | -0.11 | 0.10 | .254 | 0.89 | 0.74 | 1.08 |
| Socio economic status | -0.13 | 0.03 | <.001 | 0.88 | 0.83 | 0.93 | -0.12 | 0.04 | <.001 | 0.89 | 0.83 | 0.95 | -0.03 | 0.03 | .411 | 0.97 | 0.92 | 1.04 |
| Attendance frequency |  |  |  |  |  |  | 0.27 | 0.06 | <.001 | 1.31 | 1.18 | 1.47 | 0.32 | 0.05 | <.001 | 1.37 | 1.25 | 1.51 |
| Fandom |  |  |  |  |  |  | 0.13 | 0.09 | .135 | 1.14 | 0.96 | 1.36 |  |  |  |  |  |  |
| Team identification |  |  |  |  |  |  | -0.07 | 0.09 | .419 | 0.93 | 0.78 | 1.11 |  |  |  |  |  |  |
| Team fusion |  |  |  |  |  |  | 0.28 | 0.17 | .111 | 1.32 | 0.94 | 1.86 |  |  |  |  |  |  |
| Consumed drugs |  |  |  |  |  |  |  |  |  |  |  |  | 0.35 | 0.15 | .017 | 1.42 | 1.07 | 1.89 |
| Consumed drugs at venues |  |  |  |  |  |  |  |  |  |  |  |  | 1.57 | 0.25 | <.001 | 4.78 | 2.92 | 7.83 |
| Model summary | χ^2^(4) = 230.48, Nagelkerke R^2^ = .13, *p* <.001 | | | | | | χ^2^(8) = 200.97, Nagelkerke R^2^ = .13, *p* <.001 | | | | | | χ^2^(7) = 279.16, Nagelkerke R^2^ = .15, *p* <.001 | | | | | |
| *Note.* Country 1 = US, Gender 1 = Male, Team fusion 1 = Fused, Consumed drugs 1 = Yes, Consumed drugs at games 1 = Yes. | | | | | | | | | | | | | | | | | | |

| **Table 3.2**  Results of Logistic Binary Regression Models Predicting Self-Reported Drug Use Anywhere (Model 1), Self-Reported Drug Use at Games (Model 2), and Observed Drug Use At games (Model 3) Based on Sports And Demographic Characteristics Among US Fans | | | | | | | | | | | | | | | | | | |
| --- | --- | --- | --- | --- | --- | --- | --- | --- | --- | --- | --- | --- | --- | --- | --- | --- | --- | --- |
| Parameter | Model 1: Consumed drugs anywhere | | | | | | Model 2: Consumed drugs at games | | | | | | Model 3: Observed drugs at game | | | | | |
|  |  |  |  |  | 95% OR CI | |  |  |  |  | 95% OR CI | |  |  |  |  |  |  |
| Parameter | B | SE | *p* | OR | Lower | Upper | B | SE | *p* | OR | Lower | Upper | B | SE | *p* | OR | Lower | Upper |
| Baseball | -0.08 | 0.16 | .593 | 0.92 | 0.674 | 1.253 | 0.03 | 0.18 | .865 | 1.03 | 0.726 | 1.463 | 0.31 | 0.18 | .079 | 1.37 | 0.964 | 1.947 |
| Basketball | 0.12 | 0.15 | .450 | 1.12 | 0.830 | 1.521 | -0.05 | 0.19 | .783 | 0.95 | 0.660 | 1.368 | 0.25 | 0.18 | .155 | 1.29 | 0.909 | 1.826 |
| American Football | 0.05 | 0.15 | .732 | 1.05 | 0.779 | 1.427 | 0.20 | 0.18 | .273 | 1.22 | 0.857 | 1.728 | 0.64 | 0.18 | <.001 | 1.90 | 1.323 | 2.723 |
| Age | -0.01 | 0.00 | .060 | 0.99 | 0.982 | 1.000 | -0.01 | 0.01 | .302 | 0.99 | 0.984 | 1.005 | -0.01 | 0.01 | .191 | 0.99 | 0.983 | 1.003 |
| Gender | 0.20 | 0.11 | .075 | 1.22 | 0.980 | 1.521 | 0.19 | 0.13 | .150 | 1.21 | 0.934 | 1.558 | -0.29 | 0.13 | .030 | 0.75 | 0.579 | 0.973 |
| Socio economic status | -0.11 | 0.03 | .002 | .90 | 0.841 | 0.960 | -0.08 | 0.04 | .040 | 0.92 | 0.855 | 0.996 | 0.01 | 0.04 | .823 | 1.01 | 0.932 | 1.093 |
| Attendance frequency |  |  |  |  |  |  | 0.24 | 0.06 | <.001 | 1.27 | 1.121 | 1.430 | 0.27 | 0.07 | <.001 | 1.31 | 1.147 | 1.487 |
| Fandom |  |  |  |  |  |  | 0.11 | 0.10 | .256 | 1.12 | 0.923 | 1.352 |  |  |  |  |  |  |
| Team identification |  |  |  |  |  |  | -0.06 | 0.10 | .542 | 0.94 | 0.774 | 1.144 |  |  |  |  |  |  |
| Team fusion |  |  |  |  |  |  | 0.08 | 0.20 | .703 | 1.08 | 0.728 | 1.600 |  |  |  |  |  |  |
| Consumed drugs |  |  |  |  |  |  |  |  |  |  |  |  | 0.17 | 0.18 | .334 | 1.19 | 0.838 | 1.684 |
| Consumed drugs at venues |  |  |  |  |  |  |  |  |  |  |  |  | 1.53 | 0.27 | <.001 | 4.61 | 2.710 | 7.851 |
| Model summary | χ^2^ (6) = 19.47, Nagelkerke R^2^ = .02, *p* = .003 | | | | | | χ^2^ (10) = 30.07, Nagelkerke R^2^ = .03, *p* <.001 | | | | | | χ^2^ (9) = 116.02, Nagelkerke R^2^ = .11, *p* <.001 | | | | | |
| *Note.* Gender 1 = Male, Team fusion 1 = Fused, Consumed drugs 1 = Yes, Consumed drugs at games 1 = Yes. | | | | | | | | | | | | | | | | | | |

| **Table 3.3**  Results of Logistic Binary Regression Models Predicting Self-Reported Drug Use Anywhere (Model 1), Self-Reported Drug Use at Games (Model 2), and Observed Drug Use at Games (Model 3) Based on Sports and Demographic Characteristics Among UK Sports Fans | | | | | | | | | | | | | | | | | | |
| --- | --- | --- | --- | --- | --- | --- | --- | --- | --- | --- | --- | --- | --- | --- | --- | --- | --- | --- |
| Parameter | Model 1: Consumed drugs anywhere | | | | | | Model 2: Consumed drugs at games | | | | | | Model 3: Observed Drugs at game | | | | | |
|  |  |  |  |  | 95% OR CI | |  |  |  |  | 95% OR CI | |  |  |  |  | 95% OR CI | |
| Parameter | B | SE | p | OR | Lower | Upper | B | SE | p | OR | Lower | Upper | B | SE | p | OR | Lower | Upper |
| Rugby | 0.97 | 0.25 | <.001 | 2.64 | 1.63 | 4.28 | -0.39 | 0.30 | .199 | 0.68 | 0.37 | 1.23 | 0.40 | 0.16 | .012 | 1.49 | 1.09 | 2.03 |
| Soccer | 0.81 | 0.25 | .001 | 2.24 | 1.38 | 3.64 | -1.27 | 0.41 | .002 | 0.28 | 0.13 | 0.63 | 1.02 | 0.17 | <.001 | 2.76 | 1.97 | 3.87 |
| Age | -0.05 | 0.01 | <.001 | 0.95 | 0.94 | 0.97 | -0.04 | 0.01 | <.001 | 0.96 | 0.94 | 0.98 | -0.02 | 0.01 | <.001 | 0.98 | 0.97 | 0.99 |
| Gender | 0.61 | 0.23 | .009 | 1.84 | 1.16 | 2.90 | 0.16 | 0.32 | .616 | 1.18 | 0.63 | 2.21 | 0.21 | 0.16 | .169 | 1.24 | 0.91 | 1.68 |
| Socio economic status | -0.16 | 0.06 | .009 | 0.85 | 0.75 | 0.96 | -0.27 | 0.09 | .002 | 0.77 | 0.65 | 0.91 | -0.04 | 0.05 | .412 | 0.96 | 0.87 | 1.06 |
| Attendance frequency |  |  |  |  |  |  | 0.39 | 0.15 | .010 | 1.47 | 1.10 | 1.98 | 0.28 | 0.08 | <.001 | 1.33 | 1.14 | 1.54 |
| Fandom |  |  |  |  |  |  | 0.28 | 0.23 | .229 | 1.32 | 0.84 | 2.08 |  |  |  |  |  |  |
| Team identification |  |  |  |  |  |  | -0.20 | 0.22 | .364 | 0.82 | 0.54 | 1.26 |  |  |  |  |  |  |
| Team fusion |  |  |  |  |  |  | 0.84 | 0.36 | .019 | 2.31 | 1.15 | 4.66 |  |  |  |  |  |  |
| Consumed drugs |  |  |  |  |  |  |  |  |  |  |  |  | 0.53 | 0.27 | .048 | 1.70 | 1.004 | 2.88 |
| Consumed drugs at venues |  |  |  |  |  |  |  |  |  |  |  |  | 3.05 | 1.04 | .003 | 21.03 | 2.723 | 162.41 |
| Model summary | χ^2^ (5) = 82.29, Nagelkerke R^2^ = .13, *p* <.001 | | | | | | χ^2^ (9) = 69.43, Nagelkerke R^2^ = .16, *p* <.001 | | | | | | χ^2^ (8) = 164.81, Nagelkerke R^2^ = .19, *p* <.001 | | | | | |
| *Note.* Gender 1 = Male, Team fusion 1 = Fused, Consumed drugs 1 = Yes, Consumed drugs at games 1 = Yes. | | | | | | | | | | | | | | | | | | |

| **Table 3.4**  Crosstabulations of Substance Use, Substance Use Observations and Fan Groups in the US | | | | | | | | | | | | | | | | |
| --- | --- | --- | --- | --- | --- | --- | --- | --- | --- | --- | --- | --- | --- | --- | --- | --- |
|  | Substances used at games | | | | | | |  | Substances observed among other fans at games | | | | | | |  |
|  |  | Sports | | | | |  |  |  | Sports | | | |  |  |  |
|  |  | Baseball | Basketball | American Football | Ice Hockey | χ^2^ (*p value)* | Cramer’s V |  |  | Baseball | Basketball | American Football | Ice Hockey | χ^2^ (*p value)* | Cramer’s V | ASR |
| Cocaine | Yes | 10 (0.1) | 9 (-0.7) | 11 (0.7) | 79 (0.0) | 0.78 (.855) | 0.05 |  | Yes | 61 (-1.3) | 67 (-0.7) | 73 (-0.3) | 79 (2.3) | 5.75 (.124) | 0.07 |  |
|  | No | 71 (-0.1) | 81 (0.7) | 65 (-0.7) | 11 (0.0) |  |  |  | No | 217 (1.3) | 221 (0.7) | 230 (0.3) | 180 (-2.3) |  |  |  |
| Cannabis | Yes | 73 (1.4) | 73 (-1.4) | 65 (0.0) | 77 (0.0) | 2.679 (.425) | 0.09 |  | Yes | 230 (1.3) | 219 (-2.0) | 252 (1.6) | 202 (-0.8) | 6.51 (.089) | 0.09 |  |
|  | No | 8 (-1.4) | 17 (1.4) | 11 (0.0) | 13 (0.0) |  |  |  | No | 48 (-1.3) | 69 (2.0) | 51 (-1.6) | 56 (0.8) |  |  |  |
| MDMA/Ecstasy/ Molly | Yes | 2 (-0.6) | 4 (-0.5) | 3 (0.2) | 3 (0.1) | 0.53 (.912) | 0.04 |  | Yes | 16 (-0.1) | 17 (0.0) | 16 (-0.5) | 17 (0.6) | 0.44 (.932) | 0.02 |  |
|  | No | 79 (0.6) | 86 (0.5) | 73 (-0.2) | 87 (-0.1) |  |  |  | No | 262 (0.1) | 271 (0.0) | 287 (0.5) | 241 (-0.6) |  |  |  |
| Ketamine | Yes | 2 (-0.5) | 5 (1.4) | 1 (1.1) | 3 (0.0) | 2.57 (.462) | 0.09 |  | Yes | 8 (0.9) | 8 (0.7) | 2 (-2.2) | 7 (0.6) | 4.66 (.199) | 0.06 |  |
|  | No | 79 (0.5) | 85 (-1.4) | 75 (-1.1) | 87 (0.0) |  |  |  | No | 270 (-0.9) | 280 (-0.7) | 301 (2.2) | 251 (-0.6) |  |  |  |
| LSD/Acid | Yes | 3 (0.7) | 0 (-1.8) | 3 (0.8) | 3 (0.5) | 3.43 (.330) | 0.10 |  | Yes | 11 (1.2) | 4 (-1.8) | 6 (1.1) | 12 (1.9) | 7.01 (.069 | 0.08 |  |
|  | No | 78 (-.07) | 90 (1.8) | 73 (1.1) | 87 (-0.5) |  |  |  | No | 267 (-1.2) | 284 (1.8) | 297 (-1.1) | 246 (-1.9) |  |  |  |
| Amphetamines | Yes | 7 (0.4) | 3 (-1.8) | 8 (1.0) | 8( 0.5) | 3.54 (.315) | 0.10 |  | Yes | 13 (-2.0) | 26 1.3) | 27 (1.2) | 17 (-0.5) | 5.40 (.145) | 0.07 |  |
|  | No | 74 (-0.4) | 87 (1.8) | 68 (-1.0) | 82 (-0.5) |  |  |  | No | 265 (2.0) | 262 (-1.3) | 276 (-1.2) | 241 (0.5) |  |  |  |
| Mephedrone | Yes | 3 (1.2) | 2 (0.1) | 0 (-1.4) | 2 (0.1) | 2.68 (.443) | 0.09 |  | Yes | 10 (1.8) | 8 (0.7) | 5 (-0.8) | 2 (-1.8) | 5.78 (.123) | 0.07 |  |
|  | No | 78 (-1.2) | 88 (-0.1) | 76 (1.4) | 88 (-0.1) |  |  |  | No | 268 (-1.8) | 280 (-0.7) | 298 (0.8) | 256 (1.8) |  |  |  |
| Codeine | Yes | 6 (0.2) | 7 (0.4) | 4 (-0.6) | 6 (-0.1) | 0.47 (.926) | 0.04 |  | Yes | 21 (0.4) | 30 (2.6) | 12 (-2.4) | 16 (0.6) | 9.84 (.020) | 0.09 | Basketball *p* = .009 |
|  | No | 75 (-0.2) | 83 (-0.4) | 72 (0.6) | 84 (0.1) |  |  |  | No | 257 (-0.4) | 258 (-2.6) | 291 (2.4) | 242 (-0.6) |  |  |  |
| Rohypnol | Yes | 1 (-0.2) | 2 (0.7) | 1 (-0.1) | 1 (-0.3) | 0.47 (.925) | 0.04 |  | Yes | 1 (-0.8 ) | 3 (0.8) | 2 (-0.1) | 2 (0.1) | 0.96 (.811) | 0.03 |  |
|  | No | 80 (0.2) | 88 (-0.7) | 75 (0.1) | 89 (0.3) |  |  |  | No | 277 (0.8) | 285 (-0.8) | 301 (1.1) | 256 (-1.1) |  |  |  |
| Spice | Yes | 4 (-0.2) | 4 (-0.4) | 4 (0.0) | 6 (0.7) | 0.48 (.923) | 0.04 |  | Yes | 21 (1.6) | 12 (-1.2) | 18 (0.3) | 12 (-0.7) | 3.64 (.303) | 0.06 |  |
|  | No | 77 (0.2) | 86 (0.4) | 72 (0.0) | 84 (-0.7) |  |  |  | No | 257 (-1.6) | 276 (1.2) | 285 (-0.3) | 246 (0.7) |  |  |  |
| Other | Yes | 1 (1.9) | 4 (-0.4) | 6 (1.1) | 7 (1.2) | 4.88 (.181) | 0.12 |  | Yes | 3 (0.4) | 3 (0.3) | 3 (0.2) | 1 (-1.0) | 0.96 (.810) | 0.03 |  |
|  | No | 80 (-1.9) | 86 (0.4) | 70 (-1.1) | 83 (-1.2) |  |  |  | No | 275 (-0.4) | 285 (-0.3) | 300 (-0.2) | 257 (1.0) |  |  |  |
| Note. Adjusted standardized residuals appear in parentheses below group frequencies. | | | | | | | | | | | | | | | | |

| **Table 3.5**  Crosstabulations of Substance Use, Substance Use Observations and Fan Groups in the UK | | | | | | | | | | | | | | |
| --- | --- | --- | --- | --- | --- | --- | --- | --- | --- | --- | --- | --- | --- | --- |
|  | Drugs used at games | | | | | |  | Drugs observed among other fans at games | | | | | |  |
|  |  |  | Sports |  |  |  |  |  |  | Sports |  |  |  |  |
|  |  | Rugby | Soccer | Cricket | χ^2^ (*p value)* | Cramer’s V |  |  | Rugby | Soccer | Cricket | χ^2^ (*p value)* | Cramer’s V | ASR |
| Cocaine | Yes | 16 | 13 | 4 | 1.03 (.597) | 0.12 |  | Yes | 134 (3.4) | 133 (-0.3) | 69 (-3.4) | 16.31 (<.001) | .16 | Rugby *p* < .001  Cricket *p* < .001 |
|  | No | 14 | 19 | 4 |  |  |  | No | 87 (-3.4) | 141 (0.3) | 100 (3.4) |  |  |  |
| Cannabis | Yes | 19 | 22 | 5 | 2.68 (.262) | 0.20 |  | Yes | 113 (-0.7) | 159 (2.0) | 82 (-1.6) | 4.47 (.107) | .08 |  |
|  | No | 11 | 10 | 3 |  |  |  | No | 106 (0.7) | 115 (-2.0) | 88 (1.6) |  |  |  |
| MDMA/Ecstasy | Yes | 2 | 3 | 0 | 0.87 (.649) | 0.11 |  | Yes | 12 (-0.4) | 24 (2.5) | 4 (-2.3) | 7.68 (.021) | .11 | Soccer *p* = .012  Cricket *p* = .021 |
|  | No | 28 | 29 | 8 |  |  |  | No | 206 (0.4) | 250 (-2.5) | 165 (2.3) |  |  |  |
| Ketamine | Yes | 2 | 3 | 0 | 0.87 (.649) | 0.11 |  | Yes | 15 (-0.1) | 22 0.9) | 9 (-1.0) | 1.18 (.553) | .04 |  |
|  | No | 28 | 29 | 8 |  |  |  | No | 203 (1.0) | 252 (-0.9) | 160 (1.0) |  |  |  |
| LSD/Acid | Yes | 1 | 2 | 0 | 0.73 (.696) | 0.10 |  | Yes | 1 (-1.7) | 6 (0.9) | 4 (0.8) | 2.91 (.234) | .07 |  |
|  | No | 29 | 30 | 8 |  |  |  | No | 217 (1.7) | 268 (-0.9) | 165 (-0.8) |  |  |  |
| Amphetamines | Yes | 2 | 0 | 0 | 2.75 (.253) | 0.20 |  | Yes | 12 (1.6) | 13 (1.1) | 0 (-3.0) | 9.12 (.010) | .12 | Cricket *p* = .003 |
|  | No | 28 | 32 | 8 |  |  |  | No | 206 (-1.6) | 261 (-1.1) | 169 (3.0) |  |  |  |
| Mephedrone | Yes | 2 | 0 | 0 | 2.75 (.253) | 0.20 |  | Yes | 2 (0.3) | 2 (-0.1) | 1 (-.03) | 0.14 (.933) | .01 |  |
|  | No | 28 | 32 | 8 |  |  |  | No | 216 (-0.3) | 272 (0.1) | 168 (0.3) |  |  |  |
| Codeine | Yes | 2 | 7 | 1 | 2.95 (.229) | 0.21 |  | Yes | 5 (-0.1) | 10 (1.7) | 1 (-1.8) | 4.16 (.125) | .08 |  |
|  | No | 28 | 25 | 7 |  |  |  | No | 213 (0.1) | (264 (-1.7) | 168 (1.8) |  |  |  |
| GHB |  |  |  |  |  |  |  | Yes | 2 (0.7) | 2 (0.3) | 0 (-1.2) | 1.45 (.484) | .05 |  |
|  |  |  |  |  |  |  |  | No | 216 (-0.7) | 272 (-0.3) | 169 (1.2) |  |  |  |
| Rohypnol |  |  |  |  |  |  |  | Yes | 0 (-1.0) | 2 (1.7) | 0 (-0.8) | 2.83 (.243) | .07 |  |
|  |  |  |  |  |  |  |  | No | 218 (1.0) | 272 (-1.7) | 169 (0.8) |  |  |  |
| Spice |  |  |  |  |  |  |  | Yes | 5 (-0.9) | 13 (1.9) | 3 (-1.2) | 3.82 (.148) | .08 |  |
|  |  |  |  |  |  |  |  | No | 213 (0.9) | 261 (-1.9) | 166 (1.2) |  |  |  |
| Other |  |  |  |  |  |  |  | Yes | 1 (-0.3) | 1 (-0.7) | 2 (1.1) | 1.28 (.527) | .04 |  |
|  |  |  |  |  |  |  |  | No | 217 (0.3) | 273 (0.7) | 167 (-1.1) |  |  |  |
| Note. Adjusted standardized residuals appear in parentheses below group frequencies.  There were not enough responses for the following items to conduct analyses for drug use: GHB, Rohypnol, Synthetic cannabinoids/ Spice, Other | | | | | | | | | | | | | | |

| **Table 3.6**  Crosstabulation for Locations of Observed Drug Use and Fan Groups in the US | | | | | | | | |
| --- | --- | --- | --- | --- | --- | --- | --- | --- |
|  |  | Sports | | | |  |  |  |
|  |  | Baseball | Basketball | American Football | Ice Hockey | χ^2^ (*p value)* | Cramer’s V | ASR |
| In bathrooms | Yes | 132 (-0.3) | 150 (1.4) | 139 (-1.1) | 125 (0) | 2.39 (.496 | .05 |  |
|  | No | 146 (0.3) | 139 (-1.4) | 165 (1.1) | 134 (0) |  |  |  |
| In the stands | Yes | 132 (1.6) | 107 (-2.4) | 145 (1.9) | 105 (-1.1) | 9.87 (.020) | .09 | Basketball *p* = .016 |
|  | No | 146 (-1.6) | 181 (2.4) | 158 (-1.9) | 155 (1.1) |  |  |  |
| In transport before the game | Yes | 76 (-1.8) | 88 (-0.5) | 108 (1.6) | 86 (0.6) | 4.83 (.185) | .07 |  |
|  | No | 202 (1.8) | 200 (0.5) | 197 (-1.6) | 173 (-0.6) |  |  |  |
| In the streets | Yes | 173 (-0.2) | 193 (1.7) | 177 (-2.0) | 166 (0.5) | 5.14 (.162) | .07 |  |
|  | No | 105 (0.2) | 96 (-1.7) | 128 (2.0) | 93 (-0.5) |  |  |  |
| Note. Adjusted standardized residuals appear in parentheses below group frequencies. | | | | | | | | |

| **Table 3.7**  Crosstabulation for Locations of Observed Drug Use and Fan Groups in the UK | | | | | | | |
| --- | --- | --- | --- | --- | --- | --- | --- |
|  |  |  | Sports |  |  |  |  |
|  |  | Rugby | Soccer | Cricket | χ^2^ (*p value)* | Cramer’s V | ASR |
| In bathrooms | Yes | 125 (1.0) | 156 (1.2) | 78 (-2.4) | 5.96 (.051) | .10 |  |
|  | No | 94 (-1.0) | 118 (-1.2) | 91 (2.4) |  |  |  |
| In the stands | Yes | 75 (1.0) | 89 (0.3) | 46 (-1.5) | 2.38 (.305) | .06 |  |
|  | No | 143 (-1.0) | 185 (-0.3) | 123 (1.5) |  |  |  |
| In transport before the game | Yes | 78 (0.9) | 101 (1.5) | 43 (-2.6) | 7.03 (.030) | .10 | Cricket *p =* .009 |
|  | No | 140 (-0.9) | 173 (-1.5) | 127 (2.6) |  |  |  |
| In the streets | Yes | 96 (-2.1) | 152 (2.6) | 80 (-0.7) | 7.04 (.030) | .10 | Rugby *p* = .036  Soccer *p* = .009 |
|  | No | 123 (2.1) | 122 (-2.6) | 89 (0.7) |  |  |  |
| Note. Adjusted standardized residuals appear in parentheses below group frequencies. | | | | | | | |

| **Table 3.8**  Crosstabulations of Motives to Consume Drugs and Cocaine/Cannabis Use Among Fans in The US | | | | | | | | | | |
| --- | --- | --- | --- | --- | --- | --- | --- | --- | --- | --- |
| Motivation |  | Cocaine | |  |  |  | Cannabis | |  |  |
|  |  | No | Yes | χ^2^ (*p value)* | Cramer’s V |  | No | Yes | χ^2^ (*p value)* | Cramer’s V |
| To experiment | Yes | 35 (-3.0) | 12 (3.0) | 9.13 (.003) | 0.17 | Yes | 8 (0.5) | 39 (-0.5) | 0.27 (.603) | 0.03 |
|  | No | 261 (3.0) | 29 (-3.0) |  |  | No | 41 (-0.5) | 249 (0.5) |  |  |
| To feel good | Yes | 239 (-0.3) | 34 (-0.3) | 0.11 (.738) | 0.02 | Yes | 38 (-0.7) | 235 (0.7) | 0.45 (.504) | 0.04 |
|  | No | 57 (-0.3) | 7 (0.3) |  |  | No | 11 (0.7) | 53 (-0.7) |  |  |
| To have a good time with friends | Yes | 212 (0.4) | 28 (-0.4) | 0.20 (.659) | 0.02 | Yes | 29 (-2.0) | 211 (2.0) | 4.05 (.044) | 0.11 |
|  | No | 84 (-0.4) | 13 (0.4) |  |  | No | 20 (2.0) | 77 (-2.0) |  |  |
| To fit in with a group I like | Yes | 27 (-3.8) | 12 (3.8) | 14.28 (<.001) | 0.21 | Yes | 9 (1.6) | 30 (-1.6) | 2.59 (.108) | 0.09 |
|  | No | 269 (3.8) | 29 (-3.8) |  |  | No | 40 (-1.6) | 258 (1.6) |  |  |
| Because of boredom | Yes | 26 (-0.7) | 5 (0.7) | 0.50 (.479) | 0.04 | Yes | 7 (1.3) | 24 (-1.3) | 1.78 (.183) | 0.07 |
|  | No | 270 (0.7) | 36 (-0.7) |  |  | No | 42 (-1.3) | 264 (1.3) |  |  |
| To increase the effects of other drugs/alcohol | Yes | 25 (-4.0) | 12 (4.0) | 15.98 (<.001) | 0.22 | Yes | 7 (0.8) | 30 (-0.8) | 0.64 (.432) | 0.04 |
|  | No | 271 (4.0) | 29 (-4.0) |  |  | No | 42 (-0.8) | 258 (0.8) |  |  |
| To decrease the effects of other drugs/alcohol | Yes | 6 (-2.7) | 4 (2.7) | 7.47 (.006) | 0.15 | Yes | 1 (-0.4) | 9 (0.4) | 0.17 (.679) | 0.02 |
|  | No | 290 (2.7) | 37 (-2.7) |  |  | No | 48 (0.4) | 279 (-0.4) |  |  |
| To be more alert/ready for a fight | Yes | 12 (-2.8) | 6 (2.8) | 7.97 (.005) | 0.15 | Yes | 5 (1.6) | 13 (-1.6) | 2.68 (.102) | 0.09 |
|  | No | 284 (2.8) | 35 (-2.8) |  |  | No | 44 (-1.6) | 275 (1.6) |  |  |
| Other | Yes | 25 (-0.8) | 5 (0.8) | 0.62 (.429) | 0.04 | Yes | 5 (0.3) | 25 (-0.3) | 0.12 (.729) | 0.02 |
|  | No | **271** (0.8) | **36** (-0.8) |  |  | No | 44 (-0.3) | 263 (0.3) |  |  |
| *Note.* Adjusted standardized residuals appear in parentheses next to group frequencies. | | | | | | | | | | |

| **Table 3.9**  Crosstabulation of Motives not to Consume Drugs at Games and Fan Groups in the US | | | | | | | |
| --- | --- | --- | --- | --- | --- | --- | --- |
|  |  | Sports | | | |  |  |
|  |  | Baseball | Basketball | American Football | Ice Hockey | χ^2^ (*p value)* | Cramer’s V |
| Risk of getting caught | Yes | 18 (1.6) | 18 (-0.3) | 16 (-1.1) | 12 (-0.1) | 3.03 (.386) | .12 |
|  | No | 26 (-1.6) | 43 (0.3) | 47 (1.1) | 28 (0.1) |  |  |
| Does not fit with watching a game | Yes | 30 (0.1) | 39 (-0.7) | 43 (0.2) | 28 (0.4) | 0.49 (.921) | .05 |
|  | No | 14 (-0.1) | 22 (0.7) | 20 (-0.2) | 12 (-0.4) |  |  |
| I’m not with people who would tolerate it | Yes | 10 (0.3) | 10 (-1.1) | 16 (1.0) | 8 (-0.2) | 1.61 (658) | .09 |
|  | No | 34 (-0.3) | 51 (1.1) | 47 (-1.0) | 32 (0.2) |  |  |
| Don’t know | Yes | 3 (0.3) | 1 (-1.6) | 6 (1.5) | 2 (0.2) | 3.68 (.298) | .13 |
|  | No | 41 (-0.3) | 60 (1.6) | 57 (-1.5) | 38 (-0.2) |  |  |
| Other reasons | Yes | 6 (-0.4) | 8 (-1.0) | 13 (1.4) | 6 (-0.6) | 2.16 (.540) | .10 |
|  | No | 38 (0.4) | 54 (1.0) | 50 (-1.4) | 34 (0.6) |  |  |
| Note. Adjusted standardized residuals appear in parentheses below group frequencies. | | | | | | | |

| **Table 3.10**  *Crosstabulations of motives to consume drugs at games and cocaine and cannabis use among UK sports fans* | | | | | | | | | | | |
| --- | --- | --- | --- | --- | --- | --- | --- | --- | --- | --- | --- |
| Drug |  | Cocaine | |  |  |  |  | Cannabis | |  |  |
|  |  | No | Yes | χ^2^ (*p value)* | Cramer’s V |  |  | No | Yes | χ^2^ (*p value)* | Cramer’s V |
| To experiment | No | 27 (-0.9) | 27 (0.9) | 0.77 (.379) | .11 |  | No | 22 (1.1) | 32 (-1.1) | 1.31 (.252) | .14 |
|  | Yes | 10 (0.9) | 6 (-0.9) |  |  |  | Yes | 4 (-1.1) | 12 (1.1) |  |  |
| To feel good | No | 13 (-0.6) | 14 (0.6) | 0.39 (.532) | .08 |  | No | 13 (1.5) | 14 (-1.5) | 2.28 (.131) | .18 |
|  | Yes | 24 (0.6) | 19 (-0.6) |  |  |  | Yes | 13 (-1.5) | 30 (1.5) |  |  |
| To have a good time with friends | No | 16 (-1.1) | 10 (1.1) | 1.25 (.263) | .13 |  | No | 10 (0.2) | 16 (-0.2) | 0.03 (.861) | .02 |
|  | Yes | 21 (1.1) | 23 (-1.1) |  |  |  | Yes | 16 (-0.2) | 28 (0.2) |  |  |
| To fit in with a group I like | No | 35 (-1.7) | 27 (1.7) | 2.81 (.094) | .20 |  | No | 22 (-0.8) | 40 (0.8) | 0.64 (.424) | .10 |
|  | Yes | 2 (1.7) | 6 (-1.7) |  |  |  | Yes | 4 (0.8) | 4 (-0.8) |  |  |
| Because of boredom | No | 34 (-0.3) | 31 (0.3) | 0.11 (.740) | .04 |  | No | 23 (-1.1) | 42 (1.1) | 1.21 (.272) | .13 |
|  | Yes | 3 (0.3) | 2 (-0.3) |  |  |  | Yes | 3 (1.1) | 2 (-1.1) |  |  |
| To increase the effects of other drugs/alcohol | No | 30 (-0.4) | 28 (0.4) | 0.17 (.676) | .05 |  | No | 22 (0.3) | 36 (-0.3) | 0.09 (.764) | .04 |
|  | Yes | 7 (0.4) | 5 (-0.4) |  |  |  | Yes | 4 (-0.3) | 8 (0.3) |  |  |
| To decrease the effects of other drugs/alcohol | No | 37 (-2.5) | 28 (2.5) | 6.04 (.014) | .29 |  | No | 21 (-3.0) | 44 (3.0) | 9.11 (.003) | .36 |
|  | Yes | 0 (2.5) | 5 (-2.5) |  |  |  | Yes | 5 (3.0) | 0 (-3.0) |  |  |
| To be more alert/ready for a fight | No | 32 (-0.5) | 32 (0.5) | 0.24 (.624) | .06 |  | No | 25 (0.1) | 42 (-0.1) | 0.02 (.889) | .02 |
|  | Yes | 2 (0.5) | 1 (-0.5) |  |  |  | Yes | 1 (-0.1) | 2 (0.1) |  |  |
| Other | No | 31 (-1.8) | 32 (1.8) | 3.37 (.066) | .22 |  | No | 24 (0.5) | 39 (-0.5) | 0.25 (.621) | .06 |
|  | Yes | 6 (1.8) | 1 (-1.8) |  |  |  | Yes | 2 (-0.5) | 5 (0.5) |  |  |
| *Note.* Adjusted standardized residuals appear in parentheses next to group frequencies. | | | | | | | | | | | |

| **Table 3.11**  Crosstabulation of motives not to consume drugs at games and fan groups among UK sports fans | | | | | | |
| --- | --- | --- | --- | --- | --- | --- |
|  |  |  | Sports |  |  |  |
|  |  | Rugby | Soccer | Cricket | χ^2^ (*p value)* | Cramer’s V |
| Risk of getting caught | No | 21 (-0.2) | 19 (0) | 13 (0.2) | 0.06 (.972) | .03 |
|  | Yes | 13 (0.2) | 11 (0) | 7 (-0.2) |  |  |
| Does not fit with watching a game | No | 3 (-0.5) | 3 (-0.2) | 3 (0.7) | 0.53 (.768) | .08 |
|  | Yes | 31 (0.5) | 27 (0.2) | 17 (-0.7) |  |  |
| I’m not with people who would tolerate it | No | 22 (-0.3) | 21 (0.5) | 13 (-0.2) | 0.23 (.890) | .05 |
|  | Yes | 12 (0.3) | 9 (-0.5) | 7 (0.2) |  |  |
| Other reasons | No | 31 (1) | 26 (0) | 16 (-1) | 1.39 (.500) | .13 |
|  | Yes | 3 (-1) | 4 (0) | 4 (1) |  |  |
| Note. Adjusted standardized residuals appear in parentheses below group frequencies. | | | | | | |

| **Table 3.12**  Linear Regression Models Predicting Support for Drug Sanctions Among UK Fans | | | | | | | | | | | |
| --- | --- | --- | --- | --- | --- | --- | --- | --- | --- | --- | --- |
|  | Sanctions for Class A drugs | | | | |  | Sanctions for Class B/C drugs | | | | |
|  |  |  | 95% OR CI | |  |  |  |  | 95% OR CI | |  |
| Parameter | B | SE | Lower | Lower | p |  | B | SE | Lower | Lower | p |
| Rugby | -.13 | .08 | -.295 | .026 | .101 |  | -.08 | .08 | -.245 | .078 | .312 |
| Soccer | .03 | .09 | -.144 | .201 | .746 |  | .02 | .09 | -.153 | .195 | .815 |
| Age | .00 | .00 | -.002 | .009 | .168 |  | .00 | .00 | -.003 | .008 | .327 |
| Gender | .01 | .08 | -.148 | .158 | .947 |  | -.05 | .08 | -.205 | .104 | .521 |
| Socioeconomic status | .02 | .02 | -.030 | .065 | .466 |  | .00 | .02 | -.050 | .046 | .929 |
| Attendance frequency | -.07 | .04 | -.152 | .010 | .086 |  | -.04 | .04 | -.126 | .037 | .283 |
| Consumed drugs | -.14 | .18 | -.493 | .205 | .419 |  | -.08 | .18 | -.435 | .270 | .646 |
| Consumed drugs at venues | -.72 | .13 | -.971 | -.478 | <.001 |  | -.78 | .13 | -1.026 | -.528 | <.001 |
| Witnessed drugs at games | -.02 | .07 | -.163 | .119 | .758 |  | -.14 | .07 | -.282 | .003 | .055 |
| Fandom | .11 | .06 | -.004 | .229 | .058 |  | .06 | .06 | -.059 | .176 | .329 |
| Team identification | .13 | .05 | .024 | .227 | .016 |  | .12 | .05 | .015 | .220 | .025 |
| Team fusion | .11 | .12 | -.125 | .349 | .354 |  | .25 | .12 | .007 | .485 | .044 |
|  |  |  |  |  |  |  |  |  |  |  |  |
| Model summary | *F*(12,1072) = 8.94, *p* <.001, R^2^ = .09 | | | | |  | *F*(12,1072) = 9.03, *p* <.001, R^2^ = .09 | | | | |
| *Note.* Gender 1 = Male, Consumed drugs 1 = Yes, Consumed drugs at venues 1 = yes, Witnessed drugs at games 1 = Yes, Team fusion 1 = fused. | | | | | | | | | | | |

| **Table 3.13**  Linear Regression Models Predicting Support for Drug Sanctions Among UK Fans | | | | | | | | | | | | | | | | | | | | | | | | | | | | | |
| --- | --- | --- | --- | --- | --- | --- | --- | --- | --- | --- | --- | --- | --- | --- | --- | --- | --- | --- | --- | --- | --- | --- | --- | --- | --- | --- | --- | --- | --- |
|  | Sanctions for Cocaine | | | | |  | Sanctions for Cannabis | | | | |  | Hallucinogens | | | | |  | Prescription drugs | | | | |  | Methamphetamine | | | | |
|  |  |  | 95% CI | |  |  |  |  | 95% CI | |  |  |  |  | 95% CI | |  |  |  |  | 95% CI | |  |  |  |  | 95% CI | |  |
| Parameter | B | SE | LL | UL | p |  | B | SE | LL | UL | p |  | B | SE | LL | UL | p |  | B | SE | LL | UL | p |  | B | SE | LL | UL | p |
| Baseball | .05 | .10 | -.148 | .242 | .639 |  | .10 | .09 | -.074 | .267 | .269 |  | .00 | .10 | -.200 | .196 | .987 |  | -.02 | .10 | -.205 | .172 | .865 |  | -.04 | .10 | -.233 | .150 | .671 |
| Basketball | .18 | .10 | -.016 | .373 | .073 |  | .22 | .09 | .048 | .390 | .012 |  | .04 | .10 | -.155 | .243 | .665 |  | .02 | .10 | -.172 | .204 | .867 |  | .12 | .10 | -.072 | .311 | .221 |
| American Football | .24 | .10 | .040 | .430 | .018 |  | .05 | .09 | -.119 | .223 | .550 |  | .13 | .10 | -.069 | .328 | .200 |  | .18 | .10 | -.008 | .368 | .061 |  | .18 | .10 | -.012 | .371 | .067 |
| Age | -.01 | .00 | -.011 | .000 | .067 |  | -.01 | .00 | -.014 | -.004 | <.001 |  | .00 | .00 | -.008 | .003 | .397 |  | -.01 | .00 | -.016 | -.005 | <.001 |  | -.01 | .00 | -.014 | -.003 | .003 |
| Gender | -.14 | .07 | -.282 | -.002 | .047 |  | -.13 | .06 | -.253 | -.007 | .038 |  | -.30 | .07 | -.447 | -.161 | <.001 |  | -.19 | .07 | -.328 | -.057 | .005 |  | -.10 | .07 | -.240 | .035 | .145 |
| Socioeconomic status | .05 | .02 | .005 | .090 | .029 |  | .07 | .02 | .028 | .102 | <.001 |  | .05 | .02 | .012 | .098 | .013 |  | .03 | .02 | -.014 | .068 | .202 |  | .03 | .02 | -.016 | .068 | .226 |
| Attendance frequency | .09 | .04 | .023 | .164 | .009 |  | .18 | .03 | .122 | .245 | <.001 |  | .13 | .04 | .061 | .204 | <.001 |  | .08 | .03 | .014 | .149 | .019 |  | .08 | .04 | .012 | .150 | .022 |
| Consumed drugs at venues | -.21 | .12 | -.445 | .020 | .074 |  | -.36 | .10 | -.564 | -.158 | <.001 |  | -.31 | .12 | -.544 | -.073 | .010 |  | -.21 | .11 | -.432 | .018 | .071 |  | -.11 | .12 | -.337 | .121 | .355 |
| Consumed drugs | -.45 | .10 | -.654 | -.254 | <.001 |  | -.28 | .09 | -.450 | -.101 | .002 |  | -.45 | .10 | -.656 | -.251 | <.001 |  | -.50 | .10 | -.692 | -.306 | <.001 |  | -.48 | .10 | -.682 | -.287 | <.001 |
| Witnessed drugs at games | -.05 | .08 | -.218 | .112 | .527 |  | -.10 | .07 | -.245 | .046 | .179 |  | -.11 | .09 | -.281 | .056 | .190 |  | -.15 | .08 | -.309 | .010 | .066 |  | -.07 | .08 | -.235 | .090 | .382 |
| Legal state |  |  |  |  |  |  | -.05 | .06 | -.171 | .070 | .410 |  |  |  |  |  |  |  |  |  |  |  |  |  |  |  |  |  |  |
| Fandom | .03 | .05 | -.073 | .136 | .555 |  | .05 | .05 | -.046 | .139 | .322 |  | .00 | .05 | -.108 | .107 | .989 |  | .06 | .05 | -.045 | .157 | .277 |  | .04 | .05 | -.064 | .142 | .458 |
| Team identification | -.17 | .05 | -.277 | -.062 | .002 |  | -.16 | .05 | -.256 | -.068 | <.001 |  | -.13 | .06 | -.242 | -.023 | .017 |  | -.10 | .05 | -.208 | -.001 | .047 |  | -.11 | .05 | -.219 | -.008 | .035 |
| Team fusion | .48 | .11 | .253 | .702 | <.001 |  | .42 | .10 | .218 | .613 | <.001 |  | .39 | .12 | .164 | .623 | <.001 |  | .15 | .11 | -.065 | .369 | .169 |  | .15 | .11 | -.072 | .370 | .187 |
|  |  |  |  |  |  |  |  |  |  |  |  |  |  |  |  |  |  |  |  |  |  |  |  |  |  |  |  |  |  |
| Model summary | *F*(13,1457) = 9.61, *p* <.001, R^2^ = .08 | | | | |  | *F*(14,1386) = 13.51, *p* <.001, R^2^ = .11 | | | | |  | *F*(13,1457) = 12.17, *p* <.001, R^2^ = .10 | | | | |  | *F*(13,1457) = 10.32, *p* <.001, R^2^ = .08 | | | | |  | *F*(13,1457) = 7.24, *p* <.001, R^2^ = .06 | | | | |
| *Note.* Gender 1 = Male, Consumed drugs 1 = Yes, Consumed drugs at venues 1 = yes, Witnessed drugs at games 1 = Yes, Legal state 1= Yes, Team fusion 1 = fused. | | | | | | | | | | | | | | | | | | | | | | | | | | | | | |

# Additional Analyses Considering Legal Cannabis Status in US States

We tested how sensitive our findings were to state legalization of cannabis, given the large proportion of drug users who exclusively consume cannabis and live in legal states (Table 4.1). First, we replicated the analyses predicting drug use excluding participants from legal states (Tables 4.2 – 4.3), and a second time, we recoded the dependent variable to **not** consider individuals who reside in legal states and who report exclusive cannabis consumption as drug users (i.e., effectively predicting illicit drug consumption only) (Tables 4.4 – 4.5). Both approaches yielded highly similar results to the models including legal cannabis users, showing significant higher likelihood of consumption by US participants, and no group differences between US spectator groups (see section 3 in the supplementary materials for model results).

| **Table 4.1**  Self-reported Drug Consumption, Including and Excluding Exclusive Cannabis Users in Legal US states | | | | | | | | | | | |
| --- | --- | --- | --- | --- | --- | --- | --- | --- | --- | --- | --- |
|  |  | Football | Rugby | Cricket | All UK fans |  | All US fans | Baseball | Basketball | American Football | Ice Hockey |
| Variable | Sample N | 361 | 361 | 363 | 1085 |  | 1471 | 364 | 376 | 373 | 358 |
|  |  |  |  |  |  |  |  |  |  |  |  |
| Witnessed other fans consume drugs | % At least once | 75.9 | 60.4 | 46.6 | 60.9 |  | 76.6 | 76.4 | 76.6 | 81.2 | 72.1 |
| Consumed drugs at all | % Yes | 17.2 | 17.7 | 7.7 | 14.2 |  | 37.0  (22.1) | 34.3 (21.4) | 40.2 (24.7) | 37.3 (22.5) | 36.3  (19.6) |
| Consumed drugs at a sports event | % At least once | 8.9 | 8.3 | 2.2 | 6.5 |  | 22.9  (14.4) | 22.3 (14.8) | 23.9 (16.0) | 20.4 (12.9) | 25.1  (19.6) |
| Note. Percentages relate to Sample Ns in the top row (i.e., all fans who took the survey).  For US fans, we additionally report % not considering exclusive cannabis users from legal states in parentheses. | | | | | | | | | | | |

| **Table 4.2**  Results of Logistic Binary Regression Models Predicting Self-Reported Drug Use Anywhere (Model 1), and Self-Reported Drug Use at games (Model 2) Based on Country and Demographic Characteristics, Excluding Participants from Legal US States. | | | | | | | | | | | | |
| --- | --- | --- | --- | --- | --- | --- | --- | --- | --- | --- | --- | --- |
| Parameter | Model 1: Consumed drugs anywhere | | | | | | Model 2: Consumed drugs at games | | | | | |
|  |  |  |  |  | 95% OR CI | |  |  |  |  | 95% OR CI | |
| Parameter | B | SE | p | OR | Lower | Upper | B | SE | p | OR | Lower | Upper |
| Country | 1.00 | 0.13 | <.001 | 2.72 | 2.12 | 3.50 | 1.27 | 0.17 | <.001 | 3.55 | 2.56 | 4.94 |
| Age | -0.03 | 0.01 | <.001 | 0.97 | 0.96 | 0.98 | -0.02 | 0.01 | <.001 | 0.98 | 0.96 | 0.99 |
| Gender | 0.43 | 0.14 | .002 | 1.54 | 1.18 | 2.01 | 0.55 | 0.18 | .002 | 1.73 | 1.22 | 2.46 |
| Socio economic status | -0.18 | 0.04 | <.001 | 0.83 | 0.77 | 0.90 | -0.18 | 0.05 | <.001 | 0.83 | 0.75 | 0.92 |
| Attendance frequency |  |  |  |  |  |  | 0.37 | 0.08 | <.001 | 1.45 | 1.24 | 1.70 |
| Fandom |  |  |  |  |  |  | 0.00 | 0.12 | .985 | 1.00 | 0.79 | 1.28 |
| Team identification |  |  |  |  |  |  | -0.08 | 0.13 | .511 | 0.92 | 0.72 | 1.18 |
| Team fusion |  |  |  |  |  |  | 0.56 | 0.24 | .018 | 1.75 | 1.10 | 2.78 |
| Model summary | χ^2^ (4) = 149.63, Nagelkerke R^2^ = .13, *p* <.001 | | | | | | χ^2^ (8) = 140.72, Nagelkerke R^2^ = .15 , *p* <.001 | | | | | |
| *Note.* Sample N = 657 (Baseball n = 156, Basketball n = 182, American Football n = 165, Ice Hockey n = 154)  Gender 1 = Male, Team fusion 1 = Fused | | | | | | | | | | | | |

| **Table 4.3**  Results of Logistic Binary Regression Models Predicting Self-Reported Drug Use Anywhere (Model 1), and Self-Reported Drug Use at Games (Model 2) Based on Sports and Demographic Characteristics Among US Fans, Excluding Participants from Legal US States. | | | | | | | | | | | | |
| --- | --- | --- | --- | --- | --- | --- | --- | --- | --- | --- | --- | --- |
| Parameter | Model 1: Consumed drugs anywhere | | | | | | Model 2: Consumed drugs at games | | | | | |
|  |  |  |  |  | 95% OR CI | |  |  |  |  | 95% OR CI | |
| Parameter | B | SE | p | OR | Lower | Upper | B | SE | p | OR | Lower | Upper |
| Baseball | 0.04 | 0.26 | .864 | 1.04 | 0.63 | 1.72 | 0.09 | 0.30 | .761 | 1.09 | 0.61 | 1.96 |
| Basketball | 0.23 | 0.24 | .329 | 1.26 | 0.79 | 2.03 | 0.20 | 0.28 | .491 | 1.22 | 0.70 | 2.12 |
| American Football | 0.36 | 0.24 | .144 | 1.43 | 0.88 | 2.31 | 0.11 | 0.30 | .727 | 1.11 | 0.61 | 2.01 |
| Age | -0.01 | 0.01 | .224 | 0.99 | 0.98 | 1.01 | -0.01 | 0.01 | .466 | 0.99 | 0.98 | 1.01 |
| Gender | 0.44 | 0.18 | .012 | 1.55 | 1.10 | 2.19 | 0.70 | 0.21 | .001 | 2.01 | 1.32 | 3.06 |
| Socio economic status | -0.18 | 0.05 | <.001 | 0.84 | 0.75 | 0.93 | -0.12 | 0.06 | .066 | 0.89 | 0.78 | 1.01 |
| Attendance frequency |  |  |  |  |  |  | 0.34 | 0.10 | <.001 | 1.40 | 1.15 | 1.71 |
| Fandom |  |  |  |  |  |  | -0.14 | 0.15 | .375 | 0.87 | 0.65 | 1.18 |
| Team identification |  |  |  |  |  |  | -0.03 | 0.16 | .841 | 0.97 | 0.71 | 1.33 |
| Team fusion |  |  |  |  |  |  | 0.23 | 0.32 | .475 | 1.25 | 0.67 | 2.34 |
| Model summary | χ^2^ (6) = 21.60, Nagelkerke R^2^ = .05 , *p* = .001 | | | | | | χ^2^ (10) = 29.11, Nagelkerke R^2^ = .07, *p* = .001 | | | | | |
| *Note.* Sample N = 657 (Baseball n = 156, Basketball n = 182, American Football n = 165, Ice Hockey n = 154)  Gender 1 = Male, Team fusion 1 = Fused | | | | | | | | | | | | |

| **Table 4.4**  Results of Logistic Binary Regression Models Predicting Self-Reported Illicit Drug Use Anywhere (Model 1), and Self-Reported Illicit Drug Use at games (Model 2) Based on Country and Demographic Characteristics, Including Participants from Legal US States | | | | | | | | | | | | |
| --- | --- | --- | --- | --- | --- | --- | --- | --- | --- | --- | --- | --- |
| Parameter | Model 1: Consumed illicit drugs anywhere | | | | | | Model 2: Consumed illicit drugs at games | | | | | |
|  |  |  |  |  | 95% OR CI | |  |  |  |  | 95% OR CI | |
| Parameter | B | SE | p | OR | Lower | Upper | B | SE | p | OR | Lower | Upper |
| Country | 0.44 | 0.11 | <.001 | 1.56 | 1.25 | 1.94 | 0.83 | 0.15 | <.001 | 2.29 | 1.71 | 3.08 |
| Age | -0.03 | 0.00 | <.001 | 0.97 | 0.96 | 0.98 | -0.02 | 0.01 | <.001 | 0.98 | 0.97 | 0.99 |
| Gender | 0.27 | 0.11 | .016 | 1.31 | 1.05 | 1.62 | 0.24 | 0.14 | .080 | 1.27 | 0.97 | 1.67 |
| Socio economic status | -0.09 | 0.03 | .005 | 0.91 | 0.85 | 0.97 | -0.07 | 0.04 | .081 | 0.93 | 0.86 | 1.01 |
| Attendance frequency |  |  |  |  |  |  | 0.29 | 0.06 | <.001 | 1.34 | 1.18 | 1.52 |
| Fandom |  |  |  |  |  |  | 0.07 | 0.10 | .505 | 1.07 | 0.88 | 1.30 |
| Team identification |  |  |  |  |  |  | -0.12 | 0.10 | .229 | 0.89 | 0.73 | 1.08 |
| Team fusion |  |  |  |  |  |  | 0.47 | 0.19 | .015 | 1.60 | 1.10 | 2.33 |
| Model summary | χ^2^ (4) = 83.94, Nagelkerke R^2^ = .05, *p* <.001 | | | | | | χ^2^ (8) = 102.22, Nagelkerke R^2^ = .08 , *p* <.001 | | | | | |
| *Note.* Country 1 = US, Gender 1 = Male, Team fusion 1 = Fused | | | | | | | | | | | | |

| **Table 4.5**  Results of Logistic Binary Regression Models Predicting Self-Reported Illicit Drug Use Anywhere (Model 1), and Self-Reported Illicit Drug Use at Games (Model 2) Based on Sports and Demographic Characteristics Among US Fans, Including Participants from Legal US States | | | | | | | | | | | | |
| --- | --- | --- | --- | --- | --- | --- | --- | --- | --- | --- | --- | --- |
| Parameter | Model 1: Consumed drugs anywhere | | | | | | Model 2: Consumed drugs at games | | | | | |
|  |  |  |  |  | 95% OR CI | |  |  |  |  | 95% OR CI | |
| Parameter | B | SE | p | OR | Lower | Upper | B | SE | p | OR | Lower | Upper |
| Baseball | 0.12 | 0.19 | .524 | 1.13 | 0.78 | 1.63 | 0.03 | 0.22 | .875 | 1.03 | 0.68 | 1.58 |
| Basketball | 0.24 | 0.18 | .188 | 1.27 | 0.89 | 1.81 | 0.08 | 0.21 | .698 | 1.09 | 0.72 | 1.64 |
| American Football | 0.20 | 0.18 | .275 | 1.22 | 0.85 | 1.75 | -0.03 | 0.22 | .878 | 0.97 | 0.63 | 1.49 |
| Age | -0.01 | 0.01 | .007 | 0.99 | 0.98 | 1.00 | -0.01 | 0.01 | .134 | 0.99 | 0.98 | 1.00 |
| Gender | 0.19 | 0.13 | .152 | 1.21 | 0.93 | 1.56 | 0.27 | 0.16 | .081 | 1.31 | 0.97 | 1.79 |
| Socio economic status | -0.05 | 0.04 | .178 | 0.95 | 0.88 | 1.02 | 0.00 | 0.05 | .959 | 1.00 | 0.91 | 1.09 |
| Attendance frequency |  |  |  |  |  |  | 0.25 | 0.07 | <.001 | 1.28 | 1.11 | 1.48 |
| Fandom |  |  |  |  |  |  | 0.00 | 0.11 | .970 | 1.00 | 0.80 | 1.26 |
| Team identification |  |  |  |  |  |  | -0.12 | 0.12 | .294 | 0.89 | 0.70 | 1.11 |
| Team fusion |  |  |  |  |  |  | 0.26 | 0.23 | .264 | 1.29 | 0.82 | 2.03 |
| Model summary | χ^2^ (6) = 14.51, Nagelkerke R^2^ = .02 , *p* = .024 | | | | | | χ^2^ (10) = 23.28, Nagelkerke R^2^ = .03, *p* = .010 | | | | | |
| *Note.* Gender 1 = Male, Team fusion 1 = Fused, Consumed drugs 1 = Yes | | | | | | | | | | | | |
